# Advances on Sensors and Diagnostics of SARS-CoV‑2 Biomarkers

**DOI:** 10.1021/acs.analchem.6c02206

**Published:** 2026-07-22

**Authors:** Jaymi January, Kaylin Januarie, Nelia Sanga, Marlon Oranzie, Oluwakemi Tovide, Onyinyechi Uhuo, Emmanuel Iwuoha

**Affiliations:** SensorLab (University of the Western Cape Sensor Laboratories), 274799 University of the Western Cape, 4th Floor Chemical Sciences Building, Robert Sobukwe Road, Bellville, Cape Town 7535, South Africa

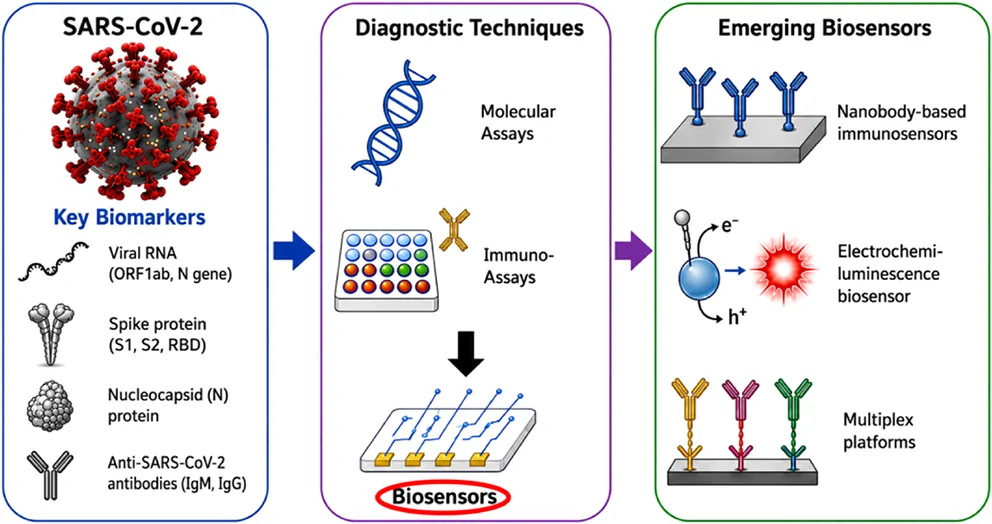

## Introduction

Communicable diseases are an ever-evolving concern. The transmission of pathogens, which can cause acute respiratory illnesses, is no exception. A viral infection is a serious global health issue that can affect people from different countries. The difficulty in detecting and treating these infections can lead to an increase in transmission and costly treatment.^1^ The coronavirus disease, known as coronavirus disease 2019 (COVID-19), was first identified in early 2020. Moreover, the global COVID-19 pandemic is the biggest public health challenge since the 1918 influenza epidemic.^2^ It has since killed over 1.8 million people and infected over 82 million individuals globally.^3^ The results of the study by the International Committee on Taxonomy of Viruses (ICTV), which was conducted based on high-throughput sequencing, revealed that COVID-19 was caused by the Severe Acute Respiratory Syndrome Coronavirus 2 (SARS-CoV-2).^4^ The World Health Organization (WHO) officially declared the COVID-19 pandemic in March 2020 due to the lack of effective treatments for the disease. The rapid emergence and evolution of the disease have created a challenging situation for various healthcare systems in different countries. The coronavirus pandemic is a type of death-dealing disease that affects people of all ages.^5^ It can affect children, the elderly, and people with certain underlying conditions. Before December 2019, six types of coronaviruses were likewise known to cause severe illness and gastrointestinal symptoms.^2^ Two of these, namely Severe Acute Respiratory Syndrome (SARS) and Middle East Respiratory Syndrome (MERS), caused severe illness and were responsible for the SARS (2003) and MERS (2012) pandemics, which now include COVID-19, the three significant developments in the history of the coronavirus pandemic.^6^ The first one, SARS, had a mortality rate of 10%, then the MERS coronavirus outbreak had a mortality rate of 37%, and, cumulatively, they both caused roughly 10,000 cases.^7^ The third pandemic (COVID-19) exhibited higher infectivity and virulence than the previous two.^4^ In addition to SARS and MERS, as mentioned, four other coronaviruses caused severe illness and were responsible for the outbreak in China in 2002.^2^ Additionally, due to genetic modification, different variants of the SARS-CoV-2 disease have emerged.^8^ Some of these cause severe illness and can trigger a high infection rate. On the other hand, others cause relatively mild symptoms and can cause an unusually high number of infections. Like other respiratory diseases, the main mode of transmission of the SARS-CoV-2 virus is through large droplets.^9^ However, it can also be transmitted through contact with contaminated objects and aerosols of varying sizes. Thus, the pandemic was caused by the emergence of an airborne transmission vector, which can easily infect people who do not have symptoms.^10^ Moreover, it defied traditional containment measures. It is expected that the disease will continue to affect a wide population for several years. Many countries have introduced effective strategies to combat the spread of the coronavirus disease by identifying and monitoring the infected individuals.^11^ However, despite the various restrictions that have been placed on the movement of people and animals due to the emergence of the coronavirus disease, the situation is not under control. It has crippled economies in several countries.

The SARS-CoV-2 virus contains over 28 different types of proteins.^12^ Generally, the structures of these proteins are composed of the NP, which is a nucleocapsid protein and is used to package the genetic material of the virus into a helical structure. The other two proteins, the spike (S1) and the envelope (E), are then positioned inside a lipidic membrane (M) to form the outer wall of the virus.^9^ In principle, the spike protein protrudes from the viral membrane. It then activates the receptor-binding domain (RBD) of the virus to enter the host cells. This process is mediated by the spike protein’s ability to recognize the angiotensin 2 (ACE2) receptor, which is a component of the SARS-CoV-2 receptor.^13^ The RBD region of the spike protein is a major target for the neutralization of the virus. It can also be utilized as a potential target for the development of diagnostic tools. The spike protein is repeated several times on the surface of the virus. However, the exact number of times this protein is replicated is still unclear. One of the most common types of antigen tests used for COVID-19 is the nucleocapsid (N) protein. However, the S protein, as mentioned, has a different sequence homology than the other viral components, allowing it to be a more accurate target for the disease. The specificity of the analytical tool depends on the target genes that are most likely to cause the disease to spread.^4^ For instance, the E gene is a highly conserved gene among all coronaviruses. The N gene is also a cross-reactive gene that can cause the disease to spread,^14^ whereas the S gene is known to differentiate SARS-CoV-2 from other coronaviruses.^15^ Thus, the S gene plays an important role in determining the degree to which the disease is infectious and spreads. Unlike the coronaviruses that emerged earlier, the fatal cases of the SARS-CoV-2 strain were not caused by a respiratory illness. Instead, they triggered multi-organ failure syndrome.^2^ The development of multi-organ failure syndrome was possibly caused by the proliferation of ACE-2. During their life cycle, the virus can develop drug targets that are related to the development of novel coronaviruses. Some of these include non-structural proteins that are like those of other coronaviruses. Compared to a previous pandemic, COVID-19 has been more contagious due to the spike protein of SARS-CoV-2. As mentioned, this protein shows affinity for ACE2, which is produced by a specific enzyme on the surface of human lung cells. The target genes of the COVID-19 pandemic are commonly to those of the previous two pandemics. Moreover, one of the most critical factors that healthcare professionals need to consider when it comes to managing the coronavirus pandemic is the estimation of the viral load. This can be done with a simple and accurate diagnostic tool.

In addition to its acute phase, it is now increasingly recognized that SARS-CoV-2 can persist in the body long after apparent clinical recovery. Persistent viral reservoirs (PVR) of SARS-CoV-2 have emerged as an important concern, particularly in the context of post-acute sequelae of COVID-19 (PASC or “long COVID”), where patients continue to experience symptoms months after infection.^16^ Several recent studies have documented that fragments of SARS-CoV-2 remain detectable in the plasma and immune cells of individuals with PASC for extended periods.^17^ This persistence suggests that SARS-CoV-2 may establish reservoirs in immune-privileged or less accessible tissues, contributing to ongoing inflammation and delayed recovery. These findings highlight the need for diagnostic approaches capable of identifying not only acute infection but also latent or persistent reservoirs.

Several different diagnostic methods have been developed to identify COVID-19. These include the use of a reverse transcription-polymerase chain reaction (RT-PCR) test,^18^ serological testing,^19^ and the detection of the virus antibodies.^20^ Serological tests are not always reliable when it comes to diagnosing patients with SARS-CoV-2 infection. This is due to the long time between the initial infection and the conversion of the virus to a bacterial form. The primary method for identifying coronaviruses is using a molecular diagnosis technique known as RT-PCR. This is because, due to the nature of the coronavirus pandemic, the use of molecular testing has become an essential tool in the fight against it. Additionally, the WHO has recommended the use of the polymerase chain reaction (PCR) method as the global standard testing strategy for the detection of coronavirus disease.^1^ Thus, due to the importance of the RT-PCR technique in the early diagnosis of SARS-CoV-2, it has been widely used in the development of various clinical trials. This method uses a combination of genetic analysis and PCR to identify the virus’s genetic material in the saliva or the naso-pelvic area.^21^ The high sensitivity of RT-PCR allows it to be used for the diagnosis of COVID-19. However, its failure rate is relatively high. This is because the contamination of the nucleic acid could lead to false-negative results.^14^ Additionally, in clinical applications, the use of single RT-PCR results can be problematic, due to the limited number of laboratories and the logistics involved; the results of the sample collection process can take up to several days. This delay in the isolation of infected carriers has been one of the main factors that have affected the containment of the pandemic. However, this method can take up to 3 h for analysis. In addition to the collection of the specimens, other steps such as the transport of the sample and the preparation of the viral RNA are also involved in the process. Besides these factors, the use of traditional RT-PCR methods can also be time-consuming and labor-intensive. This makes it difficult for large-scale tests to be performed. However, its time-consuming nature and cost-related issues can be overcome by performing rapid antigenic tests.^21^ These tests are performed using surface-associated antibodies that attach to the spike antigen. Although rapid antigenic tests are more cost-effective and faster than RT-PCR, they are still less sensitive. Thus, highlighting the need for rapid, sensitive, and selective detection techniques.

Biomarkers are often considered a kind of quantifiable label that indicates certain biological states of human bodies.^22^ Therefore, biomarkers hold enormous potential for early diagnosis and personalized therapy for disease. Biomarkers not only offer us information about existing diseases, but more importantly, they also provide individualized information regarding underlying medical conditions. By analyzing results between normal samples and patients, this information will provide morbidity, sub-clinical status, and other biological information to users in a rapid fashion. For this reason, the detection of biomarkers is important, and over the years, it has been detected using various techniques, from immunoassays to optical and electrochemical-based techniques. This article is a review of the advances in SARS-CoV-2 biomarker-based diagnostics, including the advantages of biosensor techniques over primary techniques. Nanobody, electrochemiluminescence (ECL), and multiplex-based biosensors for SARS-CoV-2 detection are also reviewed.

## Conventional/Primary Assay Techniques

### Molecular Assay

#### Polymerase Chain Reaction

One of the most widely accepted and followed diagnostic methods worldwide for the detection of SARS-CoV-2 is RT-PCR. In principle, this technique involves the conversion of viral RNA into complementary DNA (cDNA) by the reverse transcriptase enzyme. This cDNA is further amplified to several copies until the detection limit is reached through repeated heating and cooling processes. The primer specifically designed for the upstream oligonucleotide of the RNA-dependent RNA polymerase (RdRp), E gene, N gene, and Open reading Frame (ORF) gene has been used for designing RT-PCR-based kits for the detection of SARS-CoV-2 infection. Ishige *et al* ^23^ used a real-time reverse transcription-polymerase chain reaction (rRT-PCR) methodology for the simultaneous detection of two regions of the SARS-CoV-2 RNA genome (the sarbecovirus-specific E gene, the SARS-CoV-2-specific N gene), and the human ABL1 gene as an internal control. Using simplex and multiplex rRT-PCR, they compared the quantification cycle (C_q_) values of six SARS-CoV-2 RNA-positive samples and observed a good correlation between the targets. Moreover, when they used their rRT-PCR method, they detected low copies (< 25 copies/reaction) of the SARS-CoV-2 RNA. Their PCR efficiency was 2.04, and their calculated detection probability was 21 copies/reaction. They observed that the N protein was slightly more sensitive than the E protein, that the combination of the N and E proteins resulted in a higher sensitivity, and that the human ABL Proto-Oncogene 1 gene was extremely useful. Based on their results, they demonstrated that their method could detect SARS-CoV-2 with high sensitivity, use fewer reagents, and be cost-effective. However, they note that extra attention must be paid when analyzing weak positive signals due to the possibility of false-positive results. Chung *et al* ^24^ used a quantitative reverse transcription polymerase chain reaction (RT-qPCR) assay that did not require emergency use authorization (EUA) reagents for the detection of SARS-CoV-2 target genes RNA-dependent RNA polymerase (RdRp) and envelope protein (E). They were able to show that their assay had a high specificity and sensitivity for the target genes and exhibited a good analytical performance based on their assay, which was specific to the target genes and was not amplified by 13 other respiratory viruses. The assay showed good linearity, reproducibility, and repeatability with a coefficient variation of 3% and a low limit of detection (LOD) of 1 PFU/mL. They likewise compared the accuracy of their assay results with those obtained using five different COVID-19 EUA-approved kits and respiratory samples from patients with and without suspected COVID-19 and obtained a P value >0.05 and F value <5.99, which indicated good accuracy and precision for the detection of the genes. Thus, their protocol was able to show that it is possible to accurately detect COVID-19 genes using detection reagents that have not received an EUA. Yin *et al* ^25^ used a micro-PCR chip-based multifunctional platform for the rapid detection of SARS-CoV-2. They reported their method as a fast-screening technique that offered a detection limit of 10 nucleic acid copies per test in a total running time of 15 min with a throughput of 9 samples at a time. Additionally, they reported that their platform could be used as an assay of nucleic acids across 4 orders of concentration magnitude in less than 15 min. Moreover, their platform was successfully used to rapidly distinguish between negative and positive cases in a clinical environment and quantify the viral load in each sample, which was consistent with standard RT-qPCR tests. Zai *et al* ^26^ used a PCR system that was based on a gravity-driven microfluidic cartridge for the detection of not only SARS-CoV-2 but also influenza A/B and human papillomavirus 16/18. They reported that the microfluidic cartridge only contained two reaction chambers for nucleic acid lysis and amplification, which resulted in their qPCR test being passed (<30 min). They showed that using their method enabled high-throughput (12 test cartridges per test) molecular detection. They verified the analytical sensitivity and specificity of their system for respiratory pathogens and sexually transmitted diseases using SARS-CoV-2, influenza A/B RNA, and human papillomavirus 16/18 DNA samples. Their results showed a comparable detection performance to the current gold standard qPCR instrument. Moreover, their system had a very high accuracy for viral RNA/DNA detection and was validated by their Receiver-operating characteristic ROC curve analysis. Ulloa *et al* ^27^ used a simple rRT-PCR method without the use of a commercial extraction kit for the detection of SARS-CoV-2 (Orf1ab, N, and S genes) from specimens that were collected in different preservation media. They specifically compared their method to an automated protocol of nucleic acid extraction (easyMAG®) and found that the analytical results of the two procedures were similar. Within each extraction method, they collected and compared the threshold cycles (C_t_) values for each gene and observed significant changes where they noted that the N gene provided the best performance for the automated extraction, and for their extraction, the S gene was better. The results they obtained for their method agreed with those from an easyMAG® procedure. When comparing their method to a previously reported method, they saw a 3% increase in the number of positive cases detected. They evaluated the performance of the viral genes (Orf1ab, N, S), although the C_t_ value for the easyMAG® extraction was slightly better than theirs, the easyMAG® extraction required 3 times the volume of their method. Despite this, their proposed protocol was able to detect samples whose C_t_ values were at the LOD of the rRT-PCR technique. Thus, they provided a simple and inexpensive procedure suitable for laboratory detection of the SARS-CoV-2 infection. Based on the research that was obtained, it can be deduced that commercially, the RT-PCR method, due to its high accuracy and sensitivity, has been exploited and developed worldwide for the detection of COVID-19. However, despite the promising results, the prolonged time and the need for costly instruments and reagents, along with skilled personnel, are a few of the limitations of this method, which encourages researchers to search for better options. Chen *et al* ^28^ reported on a study on a SARS-CoV-2 aerosol detection platform that was enclosed in a fixed volume area (1 m^3^). They carried out their experiment in the air with wet-well cyclone air sampling and made use of the magnetic bead method (RNA extraction kit) for the extraction of the nucleic acid, whereby the platform was based on immunomagnetic nanoparticles, and droplet digital polymerase chain reaction (ddPCR) was used for the detection, as can be seen in Figure 1. They selected the specific nanoparticles because they allow for the collected particles of the virus to be collected during the sampling process. Furthermore, they used the ORF1ab gene for the primer and probe sequence. In their study, they compared the detection of the target analyte using digital PCR and the ddPCR, and from the detection results, they found that digital PCR gave a higher number of positive droplets, and the detection results differed by 10 times. They also found that when they collected the aerosol in the air and detected it using both methods, the ddPCR gave a better detection limit than the RT-PCT, which was 250 copies (102 copies/mL, concentration factor 2.5). Moreover, they obtained a sampling efficiency of 29.77% and an average extraction efficiency of 98.57%. They also extracted different concentrations of the sampling solution and then performed ddPCR, which they used to show that the consistency of the detection using ddPCR was good. Lastly, they noted that for future work, they hope to do the detection of lower concentrations of aerosols by performing multiple repeated tests.

**Figure 1:**
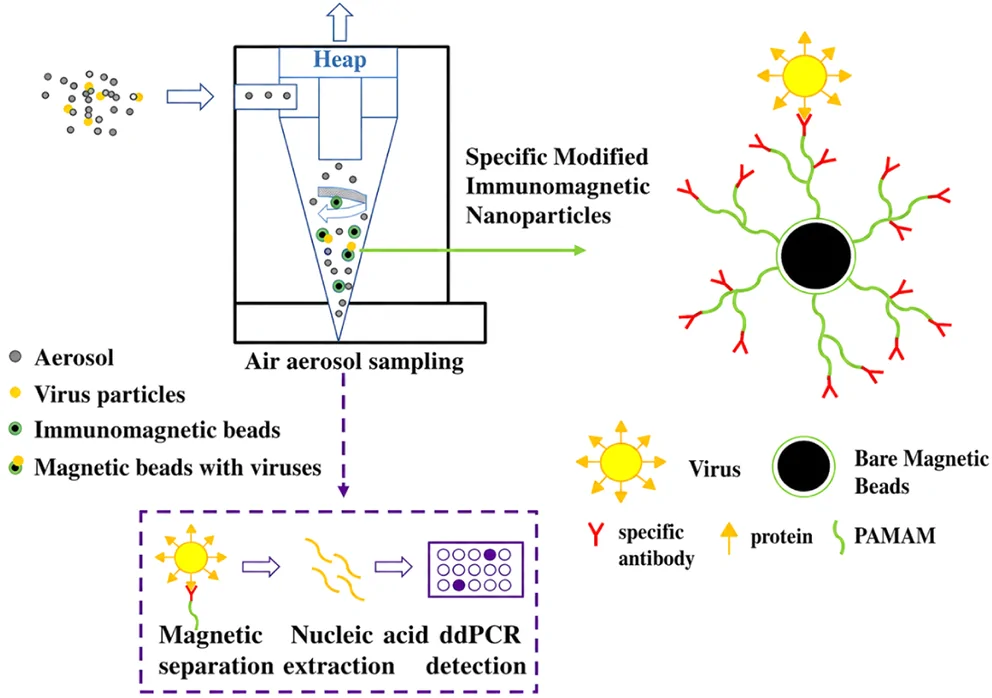
Schematic illustration of the SARS-CoV-2 aerosol detection platform reported by Chen et al. The workflow consists of four main stages: (i) collection of virus-containing aerosols using a wet-wall cyclone air sampler; (ii) adsorption and enrichment of SARS-CoV-2 particles by specific immunomagnetic nanoparticles functionalized with virus-recognition ligands; (iii) magnetic separation and concentration of the virus-bound nanoparticles from the sampling solution; and (iv) viral RNA extraction followed by nucleic acid amplification and detection using digital droplet PCR (ddPCR). The magnetic nanoparticles serve as both capture and enrichment agents, enabling efficient recovery of viral particles from aerosol samples and improving downstream detection sensitivity. Adapted from *Chinese Chemical Letters*, 2023, 34, 107701.^28^ With copyright permission from Elsevier B.V.

#### Next-Generation Genome Sequencing

The principle of the next-generation sequencing (NGS) process is that DNA or RNA is isolated and fragmented into multiple pieces instead of single fragments. Once this occurs, adapters are added for the identification and separation of sample libraries, then the libraries are sequenced and reassembled to form a genomic sequence for analysis. Nonetheless, NGS is a high-throughput technology that has been recently successfully used for the detection of the SARS-CoV-2 genome. In this context, Wang *et al*.^29^ used a nanopore targeted sequencing (NTS) for the detection of SARS-CoV-2 and other respiratory viruses simultaneously within 6–10 h. Their platform provided targeted amplification, long-read, real-time sequencing, high sensitivity, and a LOD of 10 standard plasmid copies per reaction. The results of the parallel testing of 61 COVID-19 nucleic acid samples revealed that the NTS method was more accurate and sensitive than the commercial RT-PCR technique. However, they noted that despite the excellent results they obtained, the NTS had a longer turnaround time and required more skilled operators than RT-qPCR. Thus, they highlighted that the NTS and RT-qPCR are complementary platforms. Aynaud *et al*,^30^ reported on the use of a next-generation sequencing platform “Systematic Parallel Analysis of RNA coupled to Sequencing for Covid-19 screening” (C19-SPAR-Seq) for the automated detection of SARS-CoV-2 through the targeting of RNA-dependent RNA polymerase, envelope, nucleocapsid, and two regions of the spike gene that correspond to the receptor-binding domain (RBD) and the polybasic cleavage site (PBS). They found that when their platform was coupled to the copper resistance regulator protein (coPR) on a trial cohort of hundreds of patients, it performed with a specificity of 100% and sensitivity of 9% for low viral load samples and a sensitivity of >95% for high viral load samples. Furthermore, their platform (C19-SPAR-Seq) was sensitive in detecting as low as 12.5 copies/μL of E, RBD S1, and Spbs amplicons from Twist RNA. When they compared their protocol to the current gold standard diagnostic for SARS-CoV-2 (RT-PCR), theirs was an advancement as it is readily adaptable to testing in large-scale populations. Bhoyar *et al* ^31^ used a COVIDSeq protocol that includes a multiplex-PCR, barcoded libraries, and sequencing on an NGS platform for the detection and clarification of the genetic epidemiology of SARS-CoV-2. Their analysis showed a high similarity in the detection of SARS-CoV-2 between their COVIDSeq protocol and the standard RT-PCR approach. Moreover, the results revealed that their assay had a sensitivity of 97.5% and comparable precision and accuracy to RT-PCR, and their approach had a remarkable sequencing time of 11 h and analysis time of 6 h. Their COVIDSeq protocol detected SARS-CoV-2 in samples that were previously categorized as inconclusive, pan-sarbeco, and negative when using the RT-PCR assay, which suggested that the COVIDSeq could be used as a confirmatory test. Thus, their method is compatible with different approaches for the isolation of RNA, providing wider applicability in clinical environments. NGS provides the advantage of simultaneous analysis of thousands of samples as well as high accuracy. Despite this, its applicability is limited due to it not being feasible for clinical use because of the testing duration (2 days) and its complex equipment that requires trained personnel. Moreover, genome sequencing has become more restricted as it mainly focuses on the virus’s origin and evolution. Although NGS holds many benefits, such as fast turnaround time, high sensitivity toward the detection of low-frequency variants, and the ability to provide more information on a pathogen’s gene sequence. Thus, it has a higher chance of detecting novel genes, and it can sequence a multitude of sequences simultaneously while requiring low amounts of sample input. NGS is, however, more technically demanding, requires a high cost for its operation, and because it evaluates so many genes, there is a high risk of results being inaccurate or unexpected.

#### Clustered Regularly Interspaced Short Palindromic Repeats

Clustered regularly interspaced short palindromic repeats (CRISPR) is another genome-based detection method that has shown tremendous promise as a fast (∼1 h) and accurate process for the diagnosis of COVID-19. This technique utilizes the ability of CRISPR machinery to recognize the specific genetic sequence of the target organism, and then a guide RNA attaches to a specific sequence of the genome, followed by recognition by the CRISPR enzyme. The enzyme cuts the part and generates a signal either as a fluorescence glow or as a dark band on strips; therefore, this method is sometimes referred to as “cut and detect”. Based on this working principle, Cao *et al* ^32^ used a colorimetric assay mediated with CRISPR/Cas12a and a hairpin transducer for the aggregation of gold nanoparticles (AuNPs) for the detection of SARS-CoV-2. The integration of their assay resulted in a visual detection of the E and N genes of SARS-CoV-2 in 45 min. Using the colorimetric assay for the detection of the N gene, they obtained an overall clinical sensitivity and specificity of 92.6% and 100%, respectively, for the detection of 54 clinical samples. Moreover, similar results were obtained when they detected the E gene. They noted that the assay can be used to detect other pathogens by modifying or adjusting the primers and gRNA. Broughton *et al*,^33^ used a CRISPR–Cas12–based lateral flow assay for rapid detection (< 40 min) of SARS-CoV-2 from respiratory swab RNA extracts, called SARS-CoV-2 DNA Endonuclease-Targeted CRISPR Trans Reporter (DETECTR). They observed that their DETECTR assay LOD (10 copies per μl reaction) was comparable to the U.S. Food and Drug Administration (FDA) EUA-approved complement-dependent cytotoxicity (CDC) assay for detecting SARS-CoV-2. Thus, confirming the ability of their assay to provide a visual signal. Additionally, in 36 patients with COVID-19 and 42 patients with other viral respiratory infections, their assay showed 95% positive and 100% negative predictive agreements. Park *et al*,^34^ for the first time, developed a digital-enhanced CRISPR/Cas-assisted one-pot virus detection (deCOViD) system for the detection of SARS-CoV-2 RNA and heat-inactivated SARS-CoV-2. The properties of the system allowed for qualitative detection in <15 min and quantitative detection in 30 min with a high signal-to-background ratio, broad dynamic range, and high sensitivity-down to 1 genome equivalent (GE)/μL of SARS-CoV-2 RNA and 20 GE/μL of heat-inactivated SARS-CoV-2. Based on these results, at that time, they represented one of the fastest and most sensitive CRISPR/Cas-assisted SARS-CoV-2 detection protocols. Moreover, they showed that the deCOViD could detect RNA extracts from clinical samples and further provided insight into several routes that could be used for advancing deCOViD. CRISPR tools are simple, efficient, cost-effective, and easily accessible. Even though this type of assay is more suitable for point-of-care testing than RT-PCR, there are many drawbacks limiting its application in the fight against SARS-CoV-2, such as target site restriction, increased hands-on time and human intervention, and its inability to recognize specific nucleic acids.

#### Loop-Mediated Isothermal Amplification

Loop-mediated isothermal amplification (RT-LAMP) is based on molecular detection and has the potential to replace the conventional PCR method. In principle, LAMP involves two enzymes, one to convert viral RNA to DNA and another to copy this DNA. Apart from enzymes, a set of 6 primers that are specific to a viral genome sequence is also required. Herein, DNA copying is like that in RT-PCR, but newly copied DNA strands form looped structures, which are further amplified more efficiently and rapidly than RT-PCR. Thus, González *et al.* ^35^ used a LAMP method for the detection and amplification of SARS-CoV-2 N protein. They demonstrated their method as a quantitative method that is comparable to the RT-qPCR, whereby the detection of 44 samples by their assay showed a sensitivity of 92.85%, specificity of 81.25%, and accuracy of 100% for samples that contained few copies of SARS-CoV-2 genetic material. Mautner *et al* ^36^ used an RT-LAMP-based technique for the direct detection of SARS-CoV-2 genes N and OrF8 from clinical swab samples, without the use of a laborious RNA extraction step. The assay was sensitive and selective toward the genes’ detection. Additionally, the assay showed no cross-reactivity with 20 other respiratory diseases, and they obtained a LOD of 100 copies/μL (500 copies per RT-LAMP reaction). Dao Thi *et al* ^37^ used a two-color RT-LAMP assay for the detection of the SARS-CoV-2 RNA by using a specific primer for the N gene. The detection of 768 pharyngeal swab specimens (positive for COVID) by their assay demonstrated an RT-qPCR C_t_ number of 30, a sensitivity of 97.5%, and a specificity of 99.7%. This technique is less time-consuming, cost-effective, and easy to manage, and the results can be seen with the naked eye. However, the accuracy of this technique is low, and one can perform only a few samples at a time.

### Immunoassay

#### Enzyme-Linked Immunosorbent Assay

Enzyme-linked immunosorbent assay (ELISA) is an immunological plate-based assay used to detect and measure antigens, peptides, hormones, proteins, glycoproteins, and antibodies in biological samples. The method is based on an interaction between an antigen and an antibody, whereby it captures a specific antigen in a sample by using a specific antibody and vice versa. ELISA allows for more rapid detection, lower cost, and easier operational skills when compared to RT-PCR, the gold standard method; however, the sensitivity of the ELISA is still not enough to reduce the diagnosis rate of false negatives. Therefore, researchers are developing new immunoassay techniques to improve their sensitivity and ability to detect at low concentrations in hopes of having an early diagnosis and even preventing the spread of the virus. This being the case, Yu *et al* ^38^ reported on an ELISA platform for the detection of SARS-CoV-2 NP. More specifically, their ELISA platform made use of a detection label based on an Au core-satellite nanotagulated with silica (CS@SiO_2_), which also provided a strong Surface-enhanced Raman Scattering (SERS) signal. They used silica to improve stability as well as allow for the modification of the surface so that antibodies could be anchored. Furthermore, the combination of the SERS and the ELISA increases the reproducibility and detection sensitivity, this is because in the nanogaps between NPs, the electromagnetic field is intensified. Herein, they used mouse anti-SARS-CoV-2 nucleoprotein monoclonal antibody clones and produced a sandwich-type immunocomplex using 0659Ab as the capture antibody and 0658Ab as the detection antibody. They also reduced the cost of the assay by instead of using the conventional 96-well plastic plate assay, implementing the SERS assay technology on a 384-well glass plate, which required a lower amount of the SARS-CoV-2 sample. Then, through Raman mapping after optimizing their conditions, their method obtained a relative standard deviation (RSD) value of 3.49%, showing good reproducibility. Furthermore, they analyzed the performance results of their platform and calculated a LOD of 8.81 PFU/mL, which they reported to be about 10 times better than the conventional ELISA. They also tested the specificity of their method to SARS-CoV-2 and found that only their target virus was detected amongst other viruses such as influenza A/H3N2, A/H1N1, and B. Lastly, they evaluated the clinical effectiveness of their method by using it on 30 clinical samples, whereby 24 were positive, and the rest were negative. They compared their results with those from commercial lateral flow assay (LFA) strips, and although the sensitivity at high concentrations was the same, their proposed ELISA method saw a drastic increase in sensitivity at lower SARS-CoV-2 concentrations, suggesting that their method can detect samples with 100% sensitivity with C_t_ values lower than 30. Thus, they were successfully able to reduce the false-negative diagnosis rate. Liu *et al* ^39^ developed a peptide-ELISA (p-ELISA) method based on antigen-antibody interactions for the detection of SARS-CoV-2. Herein, they made use of a peptide (phage expression of WNLDLSQWLPPM) that was specific to the SARS-CoV-2 S1, whereby they bonded to each other through hydrogen bonding, which they confirmed through molecular docking. Their platform was based on a sandwich-type p-ELISA where they used the Pn peptide as an antigen-sensing probe (capture probe) and anti-SAR-CoV-2 monoclonal antibodies (mAb) that were bound to the RBD region of the S protein as the detection antibody, and they also made use of biotin-tyramine as a signal amplifier and to enhance the overall sensitivity of the assay. Based on their obtained results, their p-ELISA assay could detect as low as 14 fM and 2.8 fM (0.4 pg/mL) for SARS-CoV-2 SP antigen via primary and secondary tyramide signal amplification (TSA) mode, respectively. Based on their results, they observed that the 2nd TSA sensitivity was enhanced by 100 times when compared to the direct mode (40 pg/mL) of the p-ELISA. Likewise, they compared their method to others such as aptamer-based biosensors, surface-enhanced resonance Raman scattering, LFA, nanobody-based ELISA, and molecular-imprinted biosensors and found their results to provide a superior detection performance. Furthermore, they noted that based on their results, there is a foundation for the testing of the 3^rd^, 4^th^, and 5th TSA. Lastly, they determined that their proposed p-ELISA with 2nd TSA could detect SARS-CoV-2 pseudoviruses in nasal swab samples down to 3 TCID50/mL and likewise confirmed that when compared to nanobody-ELISA, enzyme chemiluminescence paper, and reverse transcription loop-mediated isothermal amplification, their method gave a superior sensitivity for pseudoviruses. Luo *et al* ^40^ reported on an indirect ELISA method for the detection of SARS-CoV-2 IgG antibodies. They produced recombinant N-protein (antigen) in *Escherichia coli* and purified it for their assay fabrication, and they tagged the IgG antibody with horseradish peroxidase (HRP). They reported that the reason they chose the N-protein was that serological studies that were done on infected patients have shown that when compared to the S-protein, the N-protein-based ELISA provides higher sensitivity and is preserved longer in serum. To validate their method, they used serum samples collected from an RT-PCR that confirmed positive SARS-CoV-2 patients (113) with mild to severe symptoms. They also validated their method by using control sera samples (1500) that were collected from Germany from 2009 – 2015. They collected the above-mentioned types of samples 14 after the symptoms started. Their proposed ELISA method showed an 89.7% sensitivity (n = 68) and a 99.3% specificity for the SARS-CoV-2 patients’ samples. Then, for the 1500 control sera samples, they found that 10 of them tested positive in the ELISA, but 7 out of those 10 were false positives because for those 7 sera samples, no bands were detected, and no contaminations or N-proteins were recognized. However, they did further experiment and found that when they coated the ELISA with denatured N protein, the ELISA was able to detect 50% of those 7 false positives, thus increasing the sensitivity to 99.7%. Since bilirubin, triglycerides, and hemoglobin in plasma and serum samples are known to interfere with the ELISA, they used those samples for interference studies and found that the results differed by less than 20%. Furthermore, they tested the cross-reactivity of their method and obtained negative responses for the samples that they collected from patients who had antibody titers against parvovirus B19, Epstein-Barr virus, cytomegalovirus, herpes simplex virus, and human immunodeficiency viruses 1/2. They conducted follow-up studies and showed that one patient’s antibody titer decreased from the 9th day but still stayed positive for 65 days. Lastly, they observed that their results are quite comparable to past literature. Yadegari *et al* ^41^ developed an ELISA method that was based on an antigen-capture sandwich for the detection of SARS-CoV-2. They made use of antibodies, namely mouse MAbs that are specific to the N protein. The antigen (anti-NP MAbs) in their system was labeled with biotin via HRP-conjugated streptavidin. They generated 9 different mAbs (1D7, 2E5, 3F6, 1E3, 1B10, 1C3, 2G2, 1E1, and 1F6), whereby they evaluated each one of them separately and tried different combinations of them. Upon analyzing the reactivities, signal-to-noise ratios, and sensitivity of those mAbs, they determined that a combination of 3F6 (capture antibody) and 1C3 (detection antibody) resulted in the best performance. Based on this combination, their developed assay gave a LOD of 43 pg/mL. Furthermore, to ensure that their results were reliable, they made use of a larger number of samples (150 negative and 403 positive) that were collected from both the Omicron and Delta variants using throat specimens and oropharyngeal and nasopharyngeal swabs. It should be noted that they did not compare the results based on the type of respiratory specimen they used because it has already been evaluated, and the results showed they had a similar sensitivity.^42^ Upon assessment, when no C_t_ values and viral variants were considered, their assay obtained an overall sensitivity of 70.72% and specificity of 100%. Then, when they considered samples with C_t_ < 30 and still no variants, the sensitivity increased to 80.06%, and then for samples with C_t_ < 25, a further increase to 91.67% was observed.

#### Lateral Flow Immunoassay (LFIA)

This is a paper-based device that can detect the target analyte in a liquid sample. Typically, a detection label will be coupled to the analyte, and then, by using capillary force, the fluid will move through a test membrane that has the capture molecules imprinted on it. Then, depending on the type of test being done (competitive or sandwich assay), a positive or negative result will form based on the interaction between the detection and capture molecule.^43^ This is a very easy-to-use and rapid analytical method that is also low-cost. However, they do lack sensitivity and accuracy; therefore, researchers are focusing on improving these limitations. One of these cases is by Serebrennikova *et al*,^44^ who reported on a SERS-based LFIA for the detection of the SARS-CoV-2 S RBD protein. They modified the LFIA using AuNPs and SERS-nanotags, which was achieved through the immobilization of the antibodies (anti-SARS-CoV-2 spike RBD antibodies) and 4-mercaptobenzoic acid. They prepared a sandwich-type ELISA, with MAb RBDB2 as the primary antibody and the SERS-nanotag (Anti-RBDF5 antibody-labeled AuMBA) as the detection antibody. When they performed their experiment, they observed that in the presence of their target analyte, two colored bands were formed in the T-zone, and they further assessed the performance of their assay. Their proposed method provided a short assay detection time of 20 min and a detection limit of 0.1 ng/mL. They compared their developed SERS-based LFIA with ELISA and conventional LFIA (Au-NP-based) while keeping the immunoreagents constant and found that their proposed method had the advantages of both these methods and provided an improved sensitivity. Li *et al* ^45^ developed a dual-mode LFIA with colorimetric and Raman for the identification of SARS-CoV-2 N protein. Their assay was based on an iron­(II, III) oxide (Fe_3_O_4_) surface that was modified with 4-mercaptobenzoic acid embedded silver (Ag) nanoparticles with gold (Au) shells (ultrathin) to form Fe_3_O_4_–AgMBA@AuNPS via electrostatic interaction. This composite had magnetic-colorimetric-Raman properties, which allowed for pre-purification, enhanced SERS signals, as well as decreased any matrix interference during detection. Their detection was possible through a color band that formed when the N protein antibodies (trapped on the T-line) bound to their modified platform. To control the quality of the assay, they allowed the NP antibody to capture free nanomaterials onto the C-line. In the visual-based colorimetric detection mode, there are two prominent dark bands on the C- and T-lines for the positive sample and only one band on the C-line for the negative samples. Based on their results, they observed that the deepening of the color depends on the concentration of the antibody. Moreover, they compared the detection limits of both the SERS (0.08 pg/mL) and visual (10 – 8 mg/mL) detection analysis; they saw that the SERS had an improved sensitivity by 3 orders. Furthermore, when they compared their SERS-based LFIA to conventional LFIAs and Au gap-enhanced Raman nanotags,^46^ their assay showed to have superior sensitivity. They evaluated the specificity and reproducibility of their assay by adding several proteins to the serum samples, such as SARS-CoV-2 N protein, SARS-CoV-2 N protein antibody, SARS-CoV-2 S protein antibody, SARS-CoV-2 neutralizing antibody, anti-Staphylococcus aureus antibody, and anti-Salmonella typhimurium antibody, however evens in the presence of 100 – 200 fold of these proteins the assay still showed a superior signal on the T-line for the target analyte. For the reproducibility studies, they calculated the coefficient of variation values for both intra-assay and inter-assay, which were 8.0% and 11.7%, respectively. Lastly, the applicability of the assay was tested in serum samples that they collected from 107 volunteers, whereby 98 of them were vaccinated, and the rest were not. Their results showed that of the vaccinated samples, there were 18 weakly positive, 49 strongly positive, and the rest were negative, while the 9 unvaccinated samples were all negative. Thus, they were able to confirm that their assay could successfully identify antibody levels in serum samples. Lee *et al* ^47^ report on a study of a colorimetric LFI paper-based assay for the detection of SARS-CoV-2 via the S antigen. Their capture antibody was the S antibody (40150- D006), which was tagged with CBP31-BC protein (produced in *Escherichia coli*), and their detection antibody was a mouse mAb that was conjugated with AuNPs through physical absorption and electrostatic force. Moreover, they took into consideration the orientation of the antibodies on their platform. Thus, to have control over the orientation, they made use of the CBP31-BC linker to immobilize the antibodies on a cellulose membrane instead of a nitrocellulose membrane, which is typically used. By using this membrane, they also confirmed an increase in sensitivity in the prostate-specific antigen. On the cellulose membrane of the LFIA strip, the test zone consisted of the capture antibody with incubated CBP31-BC, and the control zone had the CBP31-BC on its own. Then, when they analysed their results, they saw that the concentration of the target analyte was directly proportional to the colour intensity of their test line. Thus, based on this, they were able to determine the LOD, which was 0.63 ng/mL (C_t_ > 30). Their assay also showed good reproducibility, as their calculated RSD value from their detection results was approximately 5%. Furthermore, the specificity of their assay was evaluated in the presence of three different concentrations of interfering S1 antigens from different types of coronaviruses (CoV–H229E, MERS-CoV, and SARS-CoV). Upon analysis, they saw that the highest interfering concentrations were detected at almost the same level as the blank, therefore highlighting the specificity of their assay towards their target analyte. They also confirmed that there was no significant cross-reactivity occurring between their target analyte and the interfering antigens. They also evaluated the applicability of their assay in SARS-CoV-2 virus samples that were cultured. They did this experiment in triplicate and found the response to be quite similar, where they obtained a sensitivity of 5 × 10^5^ copies/mL and could detect as low as 5 × 10^4^ copies/mL. Lastly, they performed a laboratory confirmation of their CBP31-BC-based LFIA in nasopharyngeal swab samples that were collected from 16 confirmed COVID-19 patients and 3 patients who were not positive for COVID-19; these were validated using RT-PCR. By using their proposed assay, they indeed obtained 16 positive results, and the healthy subjects obtained no positive signals, highlighting that the assay had an accuracy of 100%. They noted that they encountered weak intensities of the photographic images due to the surface of the cellulose membrane being inconsistent and having an uneven background. Thus, for future studies, they hope to improve this by making use of nanocellulose membranes.

As discussed above, conventional and primary techniques for SARS-CoV-2 detection exhibit high sensitivity and specificity; however, they rely on centralized laboratories, costly instrumentation, and skilled personnel, and they do not enable real-time or decentralized testing. These limitations underscore the need for portable, rapid, and highly sensitive diagnostic technologies that can be deployed at the point of care and are suitable for monitoring low-level viral persistence. In this context, Tables 1 and 2 summarize representative molecular (RT-qPCR, rRT-PCR, isothermal amplification, CRISPR, digital PCR, and sequencing) and immunoassay-based (ELISA and lateral flow) diagnostic approaches for SARS-CoV-2. The comparison highlights target biomarkers, analytical sensitivity, assay time, and operational constraints, illustrating the high clinical reliability of amplification-based methods and the simplicity and scalability of immunoassays, while emphasizing their shared limitations related to centralized infrastructure, viral-load dependence, and reduced suitability for detecting low-level or persistent infection.

**Table 1:** Comparative Overview of Validated RT-PCR Molecular Assays for SARS-CoV-2 Detection^a^

SARS-CoV-2biomarker	Detection technique	Detection Range	Limit of detection	Detection time	Limitations	Advantages	Ref.
Polymerase Chain Reaction							
E gene + N gene	rRT-PCR	2.5 – 2500 copies/reaction	21 copies/reaction (simplex NIID-N assay) and 25 copies/reaction (multiplex-clinical specimens)	-	Weak/late signals can risk false positives.	Detects two viral targets plus an internal control to validate specimen/extraction/amplification quality; authors state multiplexing can reduce reagent use/cost and handling time for clinical labs.	^23^
					Still requires nucleic acid extraction and real-time PCR instrumentation.		
**RdRp and E genes**	RT-qPCR	10^5^ – 10^–2^ PFU/mL	1 PFU/mL	∼90 min	Requires RNA extraction and real-time PCR instrumentation; validation work used cultured virus handled in BSL-3 for parts of the workflow.	Reported high specificity, high linearity (R² = 0.998 vs known PFU standards), and good precision.	^24^
						Demonstrates the feasibility of accurate testing without EUA reagents in their setting.	
SARS-CoV-2	PCR	Across four orders of magnitude of nucleic-acid concentration	10 nucleic acid copies per test.	15 min	-	Enables rapid screening and, when needed, quantification of viral load; ability to distinguish positive vs negative clinical samples, with quantification reported as consistent with standard RT-qPCR tests.	^25^
SARS-CoV-2	qPCR	100 – 500 copies/mL	200 copies/mL	< 30 min	LOD at 100 copies/mL drops below 95% positivity for all three respiratory targets (e.g., SARS-CoV-2 80%, Flu A 65%, Flu B 75%), so very-low-copy samples may be missed vs higher concentrations.	No auxiliary pumping hardware (gravity-driven), simplified cartridge design; high-throughput up to 12 cartridges per run; reported comparable detection performance to ABI 7500 and validated diagnostic accuracy.	^26^
**Orf1ab, NP, and SP**	rRT-PCR	-	-	≈ 47 min	Mean C_t_ values for Orf1ab and N were significantly lower with automated easyMAG® extraction than with PEG/NaCl precipitation (i.e., precipitation method can yield higher C_t_ / slightly reduced analytical performance for some targets).	Concordant classification with automated extraction for 94 positives +10 negatives; reported sensitivity 100%, specificity 100%, accuracy 100%.	^27^
					Still requires centrifugation + real-time PCR instrumentation.	Designed to be simple, inexpensive, and suitable when commercial extraction kits are scarce.	
SARS-CoV-2 aerosol	ddPCR	-	250 copies/mL	-	-	Reported sampling efficiency: 29.77% and extraction efficiency: 98.57%, supporting feasibility for aerosol detection.	^28^
Next-generation genome sequencing							
**ORF1ab**	NTS	-	10 standard plasmid copies per reaction.	6 −10 h	-	Reported specificity for SARS-CoV-2 = 100% (in comparison with five common respiratory viruses).	^29^
						In parallel testing of 61 suspected cases, NTS identified 22/61 as positive and could also monitor mutated sequences, categorize SARS-CoV-2 types, and detect other respiratory viruses.	
**E, N, and two Spike regions**	C19-SPAR-Seq	-	12.5 copies/μL	-	Requires multiplex PCR + barcoding + pooled NGS sequencing and analysis, which makes it a centralized, multi-step laboratory workflow rather than a simple single-instrument assay.	Designed for scale: capable of analyzing tens of thousands of samples in a single run. Reported diagnostic performance in their development cohort, depending on thresholding: e.g., ROC/PROC cutoff yielded 92% sensitivity, 100% specificity, 98% accuracy.	^30^
						Authors also report estimated retail material costs of ∼USD $9 (500 samples) to $6 (10,000 samples).	
SARS-CoV-2	Illumina COVIDSeq	-	-	∼6.2 h	The authors report that 16 samples that were RT-PCR positive were missed by COVIDSeq in their dataset.	Very high throughput: 1536 samples per single S4 flow cell run (752 clinical samples run in technical duplicates).	^31^
					Requires NGS + barcoding/library prep infrastructure (multi-step centralized workflow).	COVIDSeq detected SARS-CoV-2 in samples labeled inconclusive (21/35), pan-sarbeco (16/43), and RT-PCR negative (6/19), supporting use as a confirmatory/orthogonal method.	
						Enables genetic epidemiology/variant insights from the same run.	
Clustered Regularly Interspaced Short Palindromic Repeats							
**N and E genes**	Colorimetric	375 – 3,750,000 copies	N gene: reproducibly detectable at 225 copies (triplicates). E gene: consistently detectable at 300 copies.	45 min	Sensitivity is governed by RT-LAMP (the authors state this explicitly), and in clinical testing, they missed 2 positives with very low RNA (RT-qPCR Ct 35.4 and 37).	Single-temperature, visual readout, and “elimination of sophisticated equipment” (authors’ claim) make it promising for point-of-care; CRISPR recognition improves specificity relative to nonspecific colorimetric/LAMP-only approaches.	^32^
**E gene + N gene**	Cas12	-	10 copies/μL	∼30 min	Still requires RNA extraction for the validated workflow. Performance degrades in direct sample matrix: at ≥10% UTM, the estimated LOD worsened to 500 copies/μL, and at ≥20% PBS to 1,500 copies/μL.	No bulky thermocycler required.	^33^
						The authors report 90% sensitivity and 100% specificity vs. the CDC qRT-PCR on their evaluated respiratory swab set.	
						Requires both E and N to call a positive, supporting specificity in interpretation.	
SARS-CoV-2	Fluorescence	500 GE/μL (bulk assay saturated at ∼280 GE/μL	1 GE/μL	Qualitative detection <15 min; quantitative detection in 30 min	Current implementation relies on fluorescence-based detection and a digital microfluidic chip format (they explicitly discuss replacing fluorescence microscopy with phone-based detection as future work, meaning microscopy/reader dependence is a present constraint).	Combines speed + high sensitivity + broad dynamic range: faster qualitative calls (<15 min), 10× LOD improvement vs their bulk assay, and extended quantitative range.	^34^
						Also demonstrated feasibility for direct detection of heat-inactivated virus without RNA extraction at 20 GE/μL.	
Loop-mediated isothermal amplification							
**N protein**	Colorimetric	62 – 2 × 10^5^ DNA copies	62 copies	-	Reported diagnostic performance vs RT-qPCR in 44 patient RNA extracts: sensitivity 92.85%, specificity 81.25% (so specificity is noticeably lower than RT-qPCR in that dataset).	The authors state it is cost-effective and that colorimetric LAMP can be used as a quantitative method with diagnostic performance comparable to RT-qPCR (based on their hospital sample set).	^35^
**ORF8 and N gene**	Fluorescence	1000 – 0.2 copies/μL	100 copies/μL	-	In their swab comparison dataset, N-gene RT-LAMP failed for samples with RT-qPCR Cq ≥30 (0/7 for Cq 30–35; 0/5 for >35), while ORF8 retained partial detection, showing reduced sensitivity at lower viral loads.	No RNA extraction required (addresses extraction-kit shortages and simplifies workflow).	^36^
						High analytical specificity: no cross-reactivity across a panel of 21 respiratory pathogens (including SARS-CoV).	
						Designed to be transferable to POCT instruments (e.g., Genie II) for field use.	
**N gene**	Colorimetric	≥100 IVT RNA molecules/reaction	100 RNA molecules	25 – 30 min	Spurious amplification/background: after >30–35 min, negative controls frequently became yellowish, attributed to nonspecific RT-LAMP products.	Large clinical evaluation: tested on 768 pharyngeal swab RNA samples.	^37^
						Compared with RT-qPCR, RT-LAMP showed 97.5% sensitivity and 99.7% specificity for samples with C_t_ up to 30.	
					Clinical sensitivity drops above C_t_ ∼30 (RT-LAMP reliably detects up to RT-qPCR C_t_ 30; much less sensitive for Ct >30).	Also provides a scalable sequencing-based validation (LAMP-sequencing) for high-throughput result recording/confirmation.	

a Polymerase Chain Reaction = PCR, Systematic Parallel Analysis of RNA coupled to Sequencing for Covid-19 screening = C19-SPAR-Seq, real-time reverse-transcriptase polymerase-chain-reaction = rRT-PCR, Quantitative Polymerase Chain Reaction = qPCR, droplet digital PCR = ddPCR, European University Association = EUA, Biosafety Level 3 = BSL-3, Limit of detection (LOD), Severe acute respiratory syndrome coronavirus 2 = SARS-CoV-2, Open Reading Frame = orf, Nucleocapsid protein = NP, Spike protein = SP, Envelope gene = E gene, Nanopore targeted sequencing = NTS, Genome sequencing = NGS, Receiver Operating Characteristic = ROC, Clustered Regularly Interspaced Short Palindromic Repeats = CRISPR, Universal Transport Medium = UTM, Loop-mediated isothermal amplification = RT-LAMP, In vitro transcription = IVT.

**Table 2:** Performance Characteristics of Immunoassays for SARS-CoV-2^a^

SARS-CoV-2biomarker	Detection technique	Detection Range	Limit of detection	Detection time	Limitations	Advantages	Ref.
Immunoassays							
**NP**	UV/Vis(Fluorescence)	0 – 1000 PFU/mL	8.81 PFU/mL	-	Clinical sensitivity varies with viral load: in 30 clinical samples, sensitivity was 100% for C_t_ < 30 (15/15) but 55% for C_t_ > 30 (5/9).	Very low LOD (8.81 PFU/mL) and demonstrated ability to reduce false negatives at higher viral loads (C_t_ < 30).	^38^
					Requires a Raman/SERS readout setup.		
**SP**	Fluorescence	40 – 10,000 pg/mL	14 fM (2 pg/mL	-	Multi-step ELISA workflow (includes overnight coating); requires plate reader; signal amplification adds extra reagents/steps (TSA cycles).	Very low antigen LOD (down to 0.4 pg/mL/2.8 fM) and improved sensitivity vs direct mode (100× stated). Demonstrated detection in a complex matrix: pseudovirus spiked into nasal swab samples down to 3 TCID50/mL (2nd TSA).	^39^
**NP**	Indirect ELISA	-	-	-	Low sensitivity early after infection: 39.1% for sera collected 0 – 6 days after symptom onset/PCR confirmation; rises to 86.4% (7–13 d) and 89.7% (14 – 55 d).	High specificity: 99.3% (1500 pre-2015 control sera). The authors note it may help identify previous infections in vaccinated people because vaccines are Spike-based (anti-N can indicate infection).	^40^
					As a serology test, it detects past infection/immune response, not acute viral RNA.		
**NP**	ELISA	-	43.3 pg/mL	-	Sensitivity is strongly viral-load dependent: overall sensitivity 70.72% (all C_t_/variants), but 80.06% for C_t_ < 30 and 91.67% for C_t_ < 25 (all variants combined).	Specificity 100% overall; sensitivity did not significantly differ between Delta and Omicron subvariants in their dataset; large clinical validation across major variants.	^41^
Lateral flow immunoassay							
**RBD-SP**	Raman	0.1 – 10 ng/mL	0.1 ng/mL	20 min	Requires an additional device (Raman spectrometer) for SERS readout.	Combines LFIA speed with ∼×10 lower LOD vs ELISA (0.4 ng/mL) and ∼10× lower LOD vs qualitative LFIA (1 ng/mL visual) under their compared conditions. Enables quantitative antigen determination on a strip within 20 min.	^44^
					Also notes background/nonspecific binding of SERS nanotag can be present even without an analyte (seen in spectra).		
NP	Colorimetric + Raman	10^–6^ – 10^–6^ mg/mL	0.08 pg/mL	-	-	Magnetic separation for pre-purifying samples and decreasing matrix interference (authors stated rationale).	^45^
						Reported reproducibility: intra-assay CV 8.0%, inter-assay CV 11.7%. Demonstrated discrimination of positive/negative/weakly positive in 107 clinical serum samples.	
RBD-SP	Colorimetric	0.05 – 200 ng/mL	0.63 ng/mL	15 min	The authors note that weak photographic band intensity can occur due to inconsistencies/uneven background reflectance of cellulose paper, and they propose nanocellulose membranes as a future improvement.	Reported 100% accuracy in clinical evaluation (19 nasopharyngeal samples validated by RT-PCR; all positives/negatives correctly classified). Also designed for rapid point-of-care testing.	^46^

a Limit of detection (LOD), Plaque-forming unit = PFU, Severe acute respiratory syndrome coronavirus 2 = SARS-CoV-2, Open Reading Frame = orf, Nucleocapsid protein = NP, Spike protein = SP, Envelope gene = E gene, Lateral flow immunoassay = LFIA, Receptor Binding Domain = RBD, tyramine signal amplification = TSA, Polymerase Chain Reaction = PCR, Surface-enhanced Raman Scattering = SERS, Coefficient of Variation = %CV.

## Biosensing Techniques

According to the World Health Organization and the European Commission, rapid and accurate analysis of COVID-19 is one of the main strategies used to treat the disease.^3^ The development of rapid tests that can be used at the point-of-care could help prevent the spread of the disease and minimize the number of people infected. This led us to develop an electrochemical solution that can be used in clinical settings and as point-of-care devices. The advantages of electrochemical, electrical, and mechanical platforms are their ability to provide a wide range of quantitative and qualitative sensing capabilities.^21^ The rapid and sensitive detection of protein contamination during the coronavirus pandemic of 2019 has highlighted the need for robust and easy-to-use solutions. Furthermore, due to the increasing costs and complexity of traditional procedures, such as histopathology and chemotherapy, new detection protocols are being developed to address these issues. Emerging biosensors are regarded as a promising new field for the detection of diseases.^48^ They have been used by various diagnostic laboratories for their ability to identify and treat diseases.

Over the years, the field of biosensors has advanced immensely and has proven to be useful in various research areas, from disease diagnostics to the food and agriculture industry. Biosensors are analytical devices that contain a bioreceptor and a transducer. The bioreceptor is a biological compound (antibodies, enzymes, cells, nucleic acids, etc.) that attaches to a specific analyte. The transducer is a component that relays the biological response into a measurable signal.^49^ Based on the biological material being used, biosensors can be classified, namely, aptasensor (aptamer), immunosensor (antibody-antigen interaction), genosensor (DNA), and enzymatic-biosensor (enzyme), etc. Biosensors have been shown to have advantageous features over conventional detection techniques, such as having high sensitivity and specificity and being able to detect a variety of complex samples. They are also easy to use, portable, and provide real-time detection, which is a promising diagnostic method.^50^

Importantly, the analytical performance and behavior of biosensors are influenced by several critical factors, including the choice and orientation of the bioreceptor, surface immobilization strategies, signal amplification mechanisms, and the physicochemical properties of the transducer interface. Recent advances in nanotechnology have further enhanced biosensor performance through the incorporation of nanomaterials such as nanoparticles, graphene-based materials, and engineered nanobioreceptors like nanobodies, which offer improved stability, higher binding affinity, and reduced steric hindrance compared to conventional antibodies. In parallel, the development of advanced detection techniques, particularly ECL, has significantly improved signal sensitivity and signal-to-noise ratios. Collectively, these innovations play a crucial role in modulating biosensors. Thus, herein, these emerging strategies are critically explored to highlight their role in improving biosensor sensitivity, selectivity, and overall analytical performance within the context of modern diagnostic applications.

### Aptasensors

Aptasensors are classified as biosensors based on their biorecognition element being aptamers. Aptamers have short sequences of DNA or RNA artificial strands that can specifically bind to a target molecule and are often referred to as synthetic antibodies.^51^ They can mimic the characteristics of antibodies in various applications. However, they can also provide stability and can be used for harsh conditions. Their small size and unique design make them very easy to develop. Additionally, the versatility of aptamers makes them useful for the development of biosensors.

#### Electrochemical Detection Techniques

##### Voltametric Electrochemical Aptasensors

Rahmati *et al* ^52^ developed an ultrasensitive label-free electrochemical aptasensor based on copper hydroxide nanorods (Cu­(OH)_2_ NRs) for the measurement of SARS-CoV-2 spike glycoprotein.Their sensor showed satisfactory results in terms of the SARS-CoV-2 spike glycoprotein diagnosis, its wide linear range, low detection limit, selectivity, and the possibility of application in real samples. Their electrochemical results showed no significant peaks for the bare screen-printed carbon electrode (SPCE); however, once Cu­(OH)_2_ NRs were coated, a cathodic peak at −0.17 V appeared, which was due to the electroreduction of Cu­(OH)_2_ to Cu_2_O. The peak current was then significantly reduced when the aptamer was attached, indicating that it hindered electron transfer. Furthermore, the peak current was reduced with the addition of bovine serum albumin (BSA), as it blocked the residual active site. Lastly, with the addition of the SARS-CoV-2 spike glycoprotein, the cathodic peak current was reduced, indicating that the aptamer strings have captured the target molecules and thus confirmed the formation of the SARS-CoV-2 spike glycoprotein-aptamer complex. Their results showed a wide dynamic range of 0.1 fg/mL to 1.2 μg/mL with a high sensitivity of 1974.43 μA/mM.cm^2^ and a low detection limit of 0.03 ± 0.01 fg/mL of SARS-CoV-2 spike glycoprotein. Their developed aptasensors’ performance was comparable to previous reports, where they determined that they had a better detection limit and range, and thus they were able to develop the most sensitive aptasensor that can detect SARS-CoV-2 spike glycoprotein. They obtained a 4.1% RSD, indicating very good aptasensor reproducibility. Furthermore, the aptasensor stability was measured as it was incubated and stored at 4 °C for 10 days, where only a 2.5% decrease was observed, indicating its excellent stability. Lastly, the applicability of the sensor was tested in saliva and viral transport medium samples, and showed ultra-trace levels, as well as the provided aptasensor, was usable in clinical trials to detect the SARS-CoV-2 virus in real samples of sick and healthy individuals. Tabrizi *et al* ^53^ fabricated an aptasensor by using mesoporous silica film (MPSF) as a nano-container with encapsulated methylene blue (MB) and an aptamer electrochemical probe for the detection of SARS-CoV-2-RBD in a saliva sample. They obtained a signal analysis for their aptasensor that indicated that the surface coverage of the encapsulated MB inside the MPSF (MB@MPSF) was 1.9 nmol/cm^2^. Their developed sensor showed high sensitivity, stability, and selectivity, based on the following results. They obtained a wide linear range of 0.5 – 250 ng/mL of SARS-CoV-2-RBD in a saliva sample and a LOD of 0.36 ng/mL or 1.5 – 2.48 x 10^8^ copies/mL (using a different equation). The selectivity factor values of their aptasensor to 32 ng/mL SARS-CoV-2-RBD in the presence of C-reactive protein, hemagglutinin, and neuraminidase of influenza A virus were 35.9, 11.7, and 17.37, respectively, indicating the high selectivity of the proposed aptasensor. The inter-repeatability was determined by storing the aptasensor in a refrigerator (4 °C), and after 21 days, 95% of the aptasensor’s original response was retained. Additionally, they tested its reproducibility and found that there were no significant changes between the four different aptasensors that they used, which indicated that the aptamers were strongly attracted to the surface of the MPSF. Moreover, they compared the analytical performance of their developed aptasensor for the detection of SARS-CoV-2-RBD in saliva samples and found that they obtained mostly better results. However, there was research done on a label-based aptasensor that has been reported by Merkoçi’s group^54^ that had a slightly better performance but lacked in their response range. Also, the labeled aptamers were more expensive than the work reported by Tabrizi *et al*. Lastly, their analytical response was compared to the responses of the ELISA kit as a standard method, and found that the responses of their aptasensor did not show statistically significant differences from the ELISA kit. Similarly, Sari *et al* ^55^ reported on the development and optimization of an electrochemical aptasensor for detecting the RBD protein of SARS-CoV-2. The study focuses on enhancing the sensitivity and specificity of the aptasensor by utilizing an SPCE modified with AuNPs. The aptamer binds specifically to the RBD protein, is immobilized on the SPCE/AuNP surface through covalent bonding with linkers such as 3-mercaptopropionic acid (MPA). The immobilized aptamer is characterized using differential pulse voltammetry (DPV) with the ferri/ferrocyanide ([Fe­(CN)_6_]^3‑/4–^) redox system, confirming successful immobilization and electrochemical behavior. The optimized aptasensor demonstrates high sensitivity, specificity, and rapid response with a LOD of 2.63 ng/mL in a wide linear range, making it a promising tool for COVID-19 diagnostics and large-scale screening. The study highlights the potential of this electrochemical aptasensor in clinical diagnostics, leveraging the advantages of electrochemical biosensing and nanotechnology.

##### Impedimetric Electrochemical Aptasensors

Qi *et al* ^56^ developed a microfluidics-coupled aptasensor based on a microelectrode array (MEA) chip for ultrasensitive detection of the SARS-CoV-2 nucleocapsid protein, using a solid-liquid interface as an indicator. They were able to meet the real-time detection demand, with their response time being 15 s long. They achieved a linear range from 10^–5^ to 10^–2^ ng/mL, which they found to be very wide but lower than the threshold of the clinical diagnostic threshold, thus enabling dilution steps for later detection and analysis. The sensor reached an ultralow level of LOD, which was 3.16 × 10^–6^ ng/mL (3.16 fg/mL), a high sensitivity of 6369:1 (10^–2^ ng/mL: 1.57 x 10^–6^ ng/mL). Thus, based on their results, they were able to accomplish a high sensitivity and specificity for their target molecule. Moreover, to test the applicability of their developed sensor, they detected the N-protein in serum, saliva, tap water, and plasma and obtained LOD values of 1.82 x 10^–6^, 1.26 x 10^–6^ ng/mL, 9.62 x 10^–6,^ and 2.16 x 10^–6^, respectively. They concluded that the aptasensor and the detection method proved to be a competitive solution for real-time diagnosis of SARS-CoV-2. Tabrizi *et al*,^57^ for the first time, developed an electrochemical impedance spectroscopy-based aptasensor decorated with a carbon nanofiber-gold nanocomposite as a modifier to detect the SARS-CoV-2-RBD. The surface coverage of the aptamer was calculated to be 52.8 pmol/cm^2^. Their electrochemical response showed a good linear relationship in the range of 0.01 – 64 nM with a LOD of 7.0 pM or 1.05 – 1.69 x 10^5^ copies/μL (based on a different equation). They compared the analytical performance of their developed sensor to previously published work, and they found that it was better in most cases. However, there was research work that showed a lower LOD when compared to the developed sensor of Tabrizi *et al.* Their process of measuring 2019-nCoV-NP was more complicated, expensive, and time-consuming. Furthermore, they noticed that as the SARS-CoV-2-RBD interacted with the aptasensor, the R_ct_ of the aptasensor increased. Hence, the proposed method is a signal-based measurement method. They concluded that their proposed aptasensor showed very good analytical performances in terms of selectivity, sensitivity, excellent repeatability, and satisfactory long-term storage stability. Lastly, the aptasensor was able to detect SARS-CoV-2-RBD in a human saliva sample; they reported that the R_ct_ of the electrode did not change, indicating the biomaterials in the sample did not affect the electrochemical properties of the aptasensor. Although the prepared aptasensor paves the way for the design of a tool for the diagnosis of SARS-CoV-2-RBD in a saliva sample, it has some disadvantages. First, the sensitivity of the proposed aptasensor is less than that of immunosensors. Second, the use of commercially available electrodes already places the research at a disadvantage in terms of expanding novelty. However, the reproducibility of the signals recorded with the commercially available electrodes is better than that of the noncommercial ones. Then, Xiao *et al* ^58^ discussed the development of two innovative aptasensors for detecting inactivated SARS-CoV-2 virus. The first is a fiber-optic evanescent wave (FOEW) biosensor, which uses a DNA aptamer (named 6.9) that specifically binds to the inactivated virus with a low dissociation constant (K_D_ = 9.6 nM). This biosensor achieved detection within 6 min with a LOD of 740 fg/mL. The second is an electrochemical impedance aptasensor, which also employs the same aptamer and exhibits an even lower LOD of 5.1 fg/mL. Both aptasensors demonstrate high specificity and sensitivity, with LODs significantly lower than those of commercial colloidal gold test strips. Their study highlights the potential of these aptasensors for rapid and accurate on-site detection of SARS-CoV-2, emphasizing their importance in preventing pandemics and biothreats.

#### Other Detection Techniques

Jiang *et al* ^59^ developed a plasma-enhanced 2D metal-organic framework-based photoelectrochemical (PEC) aptasensor for the detection of SARS-CoV-2 spike glycoprotein by employing AuNPs/ytterbium and tetrakis­(4-carboxyphenyl)­porphyrin junction (Yb-TCPP) as photoactive materials with a specific DNA aptamer against the target molecule. They determined that the modified DNA aptamer on the surface of AuNPs/Yb-TCPP can bind with the S protein with high selectivity, thus decreasing the photocurrent of the system due to the high steric hindrance and low conductivity of the S protein. Based on their results, they developed an aptasensor that demonstrated a good response in the linear range 0.5 – 8 μg/mL and a detection limit of 72 ng/mL (S/N = 3). The proposed PEC aptasensor exhibited a comparable or even lower detection limit versus other sensors for S protein detection. Furthermore, they evaluated the anti-interference performance of their sensor by using pharyngeal swab extracts from healthy people, via a standard addition method. The recovery rates of three different samples were in the range of 104.0 – 106.0% with the RSD lower than 2.8%, suggesting that the proposed PEC aptasensor had good accuracy and reliability in the detection of S protein. Thus, their developed PEC aptasensor displayed excellent sensitivity, good stability, and anti-interference performance toward S protein detection. Wang *et al* ^60^ proposed a simple sandwich-type fluorescent aptasensor based on antibody-target-antigen interactions. The aptasensor was developed for the detection of the SARS-CoV-2 S protein on the surface of microplates and was based on isothermal hyperbranched rolling cycle amplification (HRCA). They likewise quantified SARS-CoV-2 spike pseudovirus in artificial saliva. Thus, they achieved a LOD of 89.7 fg/mL for S1 protein in the linear range of 100 fg/mL to 1 μg/mL. and a LOD of 51 TU/μL for the SARS-CoV-2 spike pseudo virus in artificial saliva. The sensitivity of the aptasensor was assessed in the presence of 10-fold dilutions of the S1 protein. They also evaluated the specificity of the aptasensor toward the S1 protein by introducing artificial saliva and four other proteins, namely, influenza hemagglutinin peptide fragment 1 (HA1), human serum albumin (HSA), BSA, and polyhistidine-tag (His). These protein concentrations were increased by 10000-fold compared to those of the S1 protein. HA1, artificial saliva, and His experienced no fluorescent peaks, while the BSA and HSA showed slight signals. Thus, they showed that their proposed sensor will not be suitable for the detection of SARS-CoV-2 in serum samples but is highly specific. They compared their obtained LOD values and linear range to published work that was based on the detection of SARS-CoV-2 (including the N-protein), and they obtained an improved analytical performance and wider linear range. They surmised that this improvement was due to the HRCA, as it provided an amplified signal. They concluded that their developed sensor was much simpler when compared to PCR, and exhibited a better analytical performance (lower LOD) than the ELISA kit. Nelia Sanga and co-workers^61^ present the development of a novel electrochemical aptasensor designed to detect the N protein of SARS-CoV-2. Their aptasensor utilizes mercaptosuccinic acid-capped tungsten telluride quantum dots (MSA-WTe_3_-QDs) immobilized on SPCE through 1-ethyl-3-(3-(dimethylamino)­propyl)­carbodiimide/*N*-hydroxysuccinimide (EDC/NHS) coupling chemistry. An amine-terminated 90-mer single-stranded DNA aptamer is then deposited on the modified SPCE to form the aptasensor. The chronocoulometric signal transduction technique is employed, resulting in a linear detection range of 1 – 9 pg/mL and a LOD of 0.08 pg/mL, which they reported was significantly lower than that of a commercial N-protein ELISA kit. Their aptasensor demonstrates high selectivity and recovery rates of 95 – 103.33% in simulated real samples, such as synthetic human serum, plasma, and urine. Their innovative approach highlights the potential of quantum dot-based aptasensors for sensitive and specific detection of SARS-CoV-2 biomarkers in various clinical and environmental samples. Then, Ma and co-workers^62^ developed an aptasensor based on ion current rectification (ICR) whereby they used a nanopipette that was modified with an aptamer for the detection of the N protein biomarker of SARS-CoV-2. They showed that their proposed sensor can be used for the improvement of other SARS-CoV-2 detection techniques, based on their low LOD (73.204 pg/mL) and broad linear range (100 pg/mL – 1 μg/mL). They evaluated the specificity of their sensor toward the N-protein by also detecting IgM, BSA, thrombin, and a mixture, where they found that the rectification performance was almost negligible, confirming the specificity of their sensor. They then also stored their proposed sensor for 7 days at 4 °C to assess the stability of their aptasensor and saw that after 7 days, there was no change in the rectification performance. They verified the applicability of their sensor by detecting the N-protein in human serum as well, and showed that the sensor obtained recovery percentages of 104 and 106% (different N-protein concentration samples). Thus, they showed that their sensor was reproducible and able to detect in human serum samples. They concluded that due to their modification of the capture probe, they were able to achieve a sensitive, stable, and capable platform for the detection of SARS-CoV-2. However, due to the low viral load and inadequate sensitivity of most aptasensors, they are not ideal for identifying SARS-CoV-2, especially in clinical specimens.

### Genosensors

Genosensors are devices based on nucleotide sequences that act as receptors for the specific biorecognition of complementary DNA/RNA target probes. The biorecognition event is commonly achieved in a sandwich-type assembly by the Watson and Crick complementarity between nitrogenous bases from receptors and target sequences in hybridization conditions.

#### Electrochemical Detection Techniques

##### Voltametric Electrochemical Genosensors

Cajigas *et al* ^63^ developed a SARS-CoV-2-specific electrochemical genosensor for detection and discrimination against other respiratory viruses, such as SARS-CoV, MERS, and HKU1. They developed their sensor via a sandwich-type manner, whereby a thiolated capture probe was immobilized via covalent bonds on top of maleimide-coated magnetic particles. The viral target sequence was hybridized between the capture probe and a biotinylated signaling probe. They used enzymatic complexes that are based on streptavidin horseradish peroxidase to link the biotinylated probe to provide an electrochemical response for the genosensor. They used 3 different enzymatic complexes that each contained different quantities of HRP molecules, namely, streptavidin-HRP, streptavidin poly-HRP20, and streptavidin poly-HRP80. They showed that when an enzyme complex contained more than one HRP molecule, a decrease from pM to fM will occur for the LOD value. However, they observed that the streptavidin-poly-HRP20 gave an optimal signaling tag with the lowest current background. The genosensor showed to reach a sensitivity of 174.4 μA/fM and a LOD of 807 fM when using streptavidin poly-HRP20 enzymatic complex. They were able to detect SARS-CoV-2 specifically and discriminate it against homologous viruses in spiked samples, as their current response results of their genosensor for the SARS-CoV-2 DNA sequence were significantly higher than that of the negative controls, the one and two base pair mismatches, as well as the ones with respiratory viruses. They likewise tested their developed genosensor by detection of the SARS-CoV-2 in conditions closer to a real scenario by studying its performance in a sample coming from the virus-cell culture. Their results indeed showed a comparable biosensor response between the SARS-CoV-2 cell culture sample and the negative control, with statistically significant differences (p-value <0.05). However, they found that the positive sample extracted from the cell culture showed a lower electrochemical signal. Thus, because at the time it was a first approximation of a real scenario, they opened the platform for further research, but still showed the potential of the genosensor as a tool for the detection of the virus. Farzin *et al* ^64^ developed a voltammetric genosensor based on the format of Au@CD core-shell NPs/graphite nanocrystals (GNC) that were functionalized with cDNA on a coated paper electrode for the detection of the SARS-CoV-2 RdRP gene. They exposed their genosensor to different concentrations of the virus gene, then, via a redox-active indicator, they monitored the hybridization between the cDNA probe and the RdRP gene using toluidine blue (TB). They observed that as they increased the RdRP concentration, there was an enhancement of the TB reduction peak current in a linear range of 50 pM – 12.00 nM. Furthermore, the LOD and limit of quantification (LOQ) were 0.15 pM and 0.50 pM, respectively. They obtained a repeatability RSD of 2.2%, which indicated that the response of the modified electrode was stable, validating that the formed GNC layer had a high capacity to increase the surface of the paper and increase its electrical conductivity. The spike-and-recovery studies showed acceptable recoveries for the sputum samples (>95%), which validated the accuracy of their method. The specificity of their genosensor was tested against different sequences, including the SARS RdRP gene, SARS-CoV-2 E gene, and SARS-CoV-2 RdRP gene. They showed that there was a slight increase in the response of their genosensor to the mixture containing the target (SARS-CoV-2 RdRP gene) and interfering genes as compared to that of the modified electrode for SARS-CoV-2 RdRP, which confirmed the selectivity of their developed genosensor toward the SARS-CoV-2 RdRP gene in the presence of SARS RdRP and SARS-CoV-2 E genes. Additionally, they found that the analytical performance of their protocol was comparable to that of related articles. However, they noticed that their genosensor, unlike others, did not suffer from limitations by background current and that their paper-based electrodes were nontoxic, lower cost, and resulted in a flexible biosensor. Thus, they concluded that the signal on their genosensor exhibited acceptable LOD, excellent selectivity, good stability, and high recovery for spiked sputum samples. Despite all the possible advantages of their protocol, the modification of their electrode involved several time-consuming steps. Farzin *et al* ^65^ developed a voltammetric genosensor based on carbon-based electrodes that were modified with silver ions in hexathia-18-crown-6 (HT18C6), which is a macrocyclic thio crown ligand and was used for the first time as an electrochemical probe for the detection of RdRP sequence, which is a specific target of SARS-CoV-2 virus. They modified the electrode further with chitosan and poly­(amidoamine) (PAMAM) dendrimer-coated silicon quantum dots to provide a larger surface area as well as amino groups for the immobilization of the DNA probe. Based on the signal-off trend, they obtained that their genosensor showed a good linear response in the concentration range 1.0 pM – 8.0 nM to the SARS-CoV-2 RdRP. Furthermore, they obtained a LOD and quantification values of 3 pM and 1.0 pM, respectively. They tested the specificity of their carbon-paste electrode (CPE)–HT18C6­(Ag)/chitosan/SiQDs@PAMAM probe toward different sequences, such as SARS-CoV-2 RdRP, SARS RdRP, and envelope protein gene. However, they found that there was a significant decrease in the peak current of silver toward the SARS-CoV-2 RdRP sequence. This indicated that the probe was selectively bound to the target sequence. The repeatability and reproducibility of their genosensor were less than 2.7% and 6.6%, respectively. Moreover, their stability results showed a negligible current decay. They showed that their genosensor could be used to determine the SARS-CoV-2 RdRP sequence in sputum samples, based on their good recovery for real sample analysis (> 95%). Lastly, they found that their work showed analytical performance that was comparable to previous research work and thus was acceptable. However, despite their many advantages, they found that their method is time-consuming and costly, and their design could potentially provide negative signals. Silva *et al* ^66^ developed a 3D-printed graphene polylactic (G-PLA) electrode modified with AuNPs for the detection of cDNA of SARS-CoV-2 and creatinine (a possible COVID-19 biomarker). Their detection strategy was based on the signal-off response of the Au on the surface of the electrode after complexation with a convolutional neural network (CNN) for the sensor and after the interaction with the cDNA strand that was immobilized on the modified sensor surface with the target sequence. The cyclic voltammetry (CV) sensing capability of the cDNA of the virus presented a linear response in the range of 1.0 – 50.0 μmol/L with a good LOD (0.30 μmol/L), and high sensitivity (0.583 μA/μmol/L). Moreover, they determined the analytical performance of their genosensor via electrochemical impedance spectroscopy (EIS) and obtained a sensitivity of 0.115 kΩ/μmol/L and detection and quantification limit values of 0.31 and 0.93 μmol/L, respectively. The reproducible results they obtained were RSD = 1.14%, n = 3, attesting to the potential of 3D-printed platforms to produce biosensors. They observed the selectivity of their method by evaluating the genosensor in the presence of a negative target (Influenza A DNA sequence). They observed that there were no significant changes in the analytical signals, and based on the data they collected, they showed that the DNA sequence from the virus can be detected with good specificity. Lastly, they demonstrated the applicability of their genosensor by analyzing the target in synthetic saliva and human serum and found that the analytical responses were obtained after 30 min, and recovery values from 96.0 – 102.0% and 97.0 – 101.0%, respectively. Additionally, they successfully detected creatinine as well and were able to do detection at low concentrations with a LOD of 0.016 mmol/L with a high sensitivity of 9.318 μA/mmol/L and provided adequate recovery values, which ranged from 94.0 – 110.0%. Thus, they proved that their developed genosensor was suitable for sample analysis and had great potential for detecting the SARS-CoV-2 virus while using simple, fast techniques and low-cost materials. Ang *et al* ^67^ developed an electrochemical genosensor for the rapid detection of the SARS-CoV-2 genomic sequences, based on an electroactive nanocarbon material, namely graphene oxide nanocolloids (GONC), which acted as both the transducing platform for immobilization and the electroactive label for signal generation. The biorecognition element they used in their research was a complementary short-stranded sequence of the RdRp genome sequences of SARS-CoV-2. They found that when the DNA probe was immobilized onto their modified electrode, there was an initial decrease in the electrochemical signal, but when the target sequence was present, the electrochemical signal was restored. Moreover, their genosensor showed to have a wide dynamic linear range and a LOD value of 186 × 10^–9^ M. Thus, based on their results, they showed that their genosensor had a high selectivity with insignificant interference for the SARS-CoV-2 target. They concluded that their proposed detection technique could be integrated into a DNA amplifier for portable and low-cost detection of the SARS-CoV-2 virus in infected patients. Malla *et al* ^68^ reported on a DNA sandwich probe-type genosensor based on a reduced graphene oxide Au-decorated magnetic nanocomposite for the detection of the SARS-CoV-2 N gene. They amplified the signal of their biotinylated detection probe 8 times by linking it with an HRP enzyme with hydrogen peroxide as the substrate and anthraquinone as the electron mediator between the HRP and the working electrode. They were able to do the detection of the N-gene by using the mechanism of the specific Watson-Crick base-pairing that occurred between the capture probe and the target gene sequence. The use of the magnetic nanocomposite was to reduce the possibility of pipette error and cross-contamination. They tested the applicability of their sensor by doing detection in saliva, urine, and serum, and found that the sensor achieved detection limits of 0.37, 0.33, and 0.19 fM, respectively. They, likewise, showed that detection in the serum sample gave the best results in terms of its sensitivity and detection limit. Moreover, they obtained a wide linear range of 5 aM – 50 pM with an assay time of 45 min. The stability of their genosensor was also evaluated by storing it at 4 °C for 3 and 13 weeks, and found that the sensor retained 90% and 80%, respectively, of the initial response. Furthermore, when they compared their platform to other sandwich-type recognition platforms, they observed that they had a lower LOD and a larger linear range. However, they noted that since their sensor was developed on a screen-printed electrode, it only had a shelf life of 2 years, as well as they could not perform any clinical trials. da Silva Catunda *et al* ^69^ proposed a photoelectrochemical genosensor based on screen-printed electrodes modified with glutathione-coated Au nanoparticles and 2D-carbonylated graphitic carbon nitride (c-*g*-C_3_N_4_), poly­(diallyl dimethylammonium) chloride (PDDA) for the detection of SARS-CoV-2 virus in real saliva samples. An increase in photocurrent and overall improvement in the detection were observed due to these materials being combined. The biomarkers they used for their sensor were capture probes DNA (cpDNA) 1A and 3A. However, the primary biomarker was the cpDNA 1A, as it was able to identify Delta variants much more efficiently. They miniaturized their sensor device by using Arduino and 3d printing. Their sensor showed excellent analytical results with LOD values of 2.2 fmol/L and 3.4 fmol/L for the cpDNA 1A and 3A, respectively. Likewise, they found their sensitivity values to be greater than those of sensors found in the literature. They used their receiver operator characteristic (ROC) curve to compare their obtained photoelectrochemical results to the results of an RT-PCR technique obtained with saliva samples and nasopharyngeal and oropharyngeal swabs. Thus, they obtained an excellent value from the ROC, where the area under the curve was 0.867 at p < 0.001. However, they note that a sandwich-type assay does provide more accuracy than their developed sensor, but since their sensor is label-free, it does not require as many modifications. They obtained an analysis time of 60 min, and because there are sensors that provide shorter analysis times, they would need to optimize their experiment conditions. They obtained a satisfactory accuracy of their sensor, as the accuracy and sensitivity of their sensor were 80% and 93.3%, respectively. Despite this, there are still various challenges that need to be overcome to develop genosensors that can effectively detect SARS-CoV-2. These include sensitivity, repeatability, nonspecific signals, and analysis time.

### Immunosensors

Immunosensors are biosensors that use antibodies as the recognition element. It fundamentally works on the principle of a stable complex being formed between the antigens and antibodies, and uses the binding event to provide a measurable signal.^70^ The antigen-antibody reaction is reversible, and the dissociation is dependent on the strength of the bond. Immunosensors have become a popular analytical instrument owing to their excellent detection performance. Moreover, recently, immunosensors have been advanced as their detection approach can be incorporated with recent digital technologies.

#### Electrochemical Detection Techniques

##### Amperometry Electrochemical Immunosensors

Eissa *et al*,^71^ for the first time, developed a carbon nanofiber-based cotton-tipped electrochemical immunosensor for the detection of the SARS-CoV-2 N protein virus antigen. In Figure 2, the schematic diagram of the immunosensor fabrication is revealed. They were able to detect the virus antigen via swabbing, followed by a competitive assay using a fixed amount of N protein antibody in the solution. Based on the electrochemical results, they observed that the (carbon nanofiber) CNF-modified electrode experienced a significant reduction in peak current enhancement due to the increase in the electrochemical surface area of the electrode, due to the CNF material. They also observed that when the N protein was immobilized onto the modified electrode, they saw a further increase in the reduction peak current, which was due to the shielding of the negatively charged carboxylic groups on the CNF surface with the antigen. Their results indicated that their developed biosensor had a good analytical performance, based on their LOD value of 0.8 pg/mL and a wide linear range from 1 – 1000 ng/mL. They further showed that their LOD value was much lower than the LOD of ELISA for the SARS-CoV-2 N protein (0.4 ng/mL). Moreover, their biosensor did not show significant cross-reactivity with other virus antigens such as influenza A and HCoV, indicating high selectivity of the method. To investigate the practical applicability of their developed cotton-tipped electrochemical immunosensor for the detection of the virus in the nasal fluid, the sensor was used to collect the nasal fluid from healthy volunteers and then subjected to the electrochemical measurement of protein on the cotton immunosensor. This indicated the success of the cotton immunosensor in collecting and detecting the virus protein with high accuracy and without significant interference from the other components of the nasal fluid. Lastly, they investigated the performance of their electrochemical biosensor for the detection of SARS-CoV-2 in clinical samples, and nasal swabs from two healthy individuals and two patients were collected and stored in universal transport medium (UTM). The positive and negative samples were confirmed by performing RT-PCR. The biosensor response to the patient samples was much lower than the normal healthy sample. These results indicate that our COVID-19 electrochemical biosensor can discriminate between patient and normal samples and show good agreement with the PCR results. Li *et al* ^72^ developed a sandwich-immunosensor based on a novel trimetallic nanozyme (PdPtRu nano dendrite) for SARS-CoV-2 antigen detection. The combination of the materials possessed many advantages, such as amplified signals, peroxidase-like activity, improved electrocatalytic activity, and providing active sites for the immobilization of the capture antibody. The sandwich-type immunosensor was formed between the capture antibody (Ab1) and a detection antibody (Ab2), which was labeled with SiO_2_ nanospheres. Thus, as the concentration of the SARS-CoV-2 antigen increased, there was a decrease in the current signal due to the inhibitory effect of nanospheres. This highlighted the feasibility of their immunosensor for the detection of the S protein, as it showed a high immune-recognition ability of the antigen and antibodies, with SiO_3_ providing a strong ability to hindrance for electron transfer. Their proposed sensor showed sensitive detection of the target analyte with a LOD of 51.74 fg/mL and a linear range of 1 pg/mL – 1 μg/mL. They investigated the specificity of their sensor for the target analyte by introducing four other human coronaviruses as interference substances (SARS-CoV, HCOV-OC43, HCOV-HKU1, and MERS-CoV) with a 100 times concentration increase. Thus, when they compared the response to SARS-CoV-2 alone to the mixture of all 5 antigens, there was only a slight difference, indicating the excellent specificity of the immunosensor toward the detection of SARS-CoV-2 antigen. They used intra- and inter-assays to assess the reproducibility of their sensor and obtained RSD values of 0.51 and 0.22%, respectively. They also tested the storage stability by storing the immunosensor at 4 °C for 1 week, 2 weeks, 3 weeks, and 4 weeks, and obtained a decrease in current response of 1, 2.42, 4.23, and 7,81%, respectively. Lastly, to investigate the practical application of their sensor, they detected the S protein in nasopharyngeal swabs from healthy people as well, and they obtained a recovery range of 96.32 – 106.7%.

**Figure 2:**
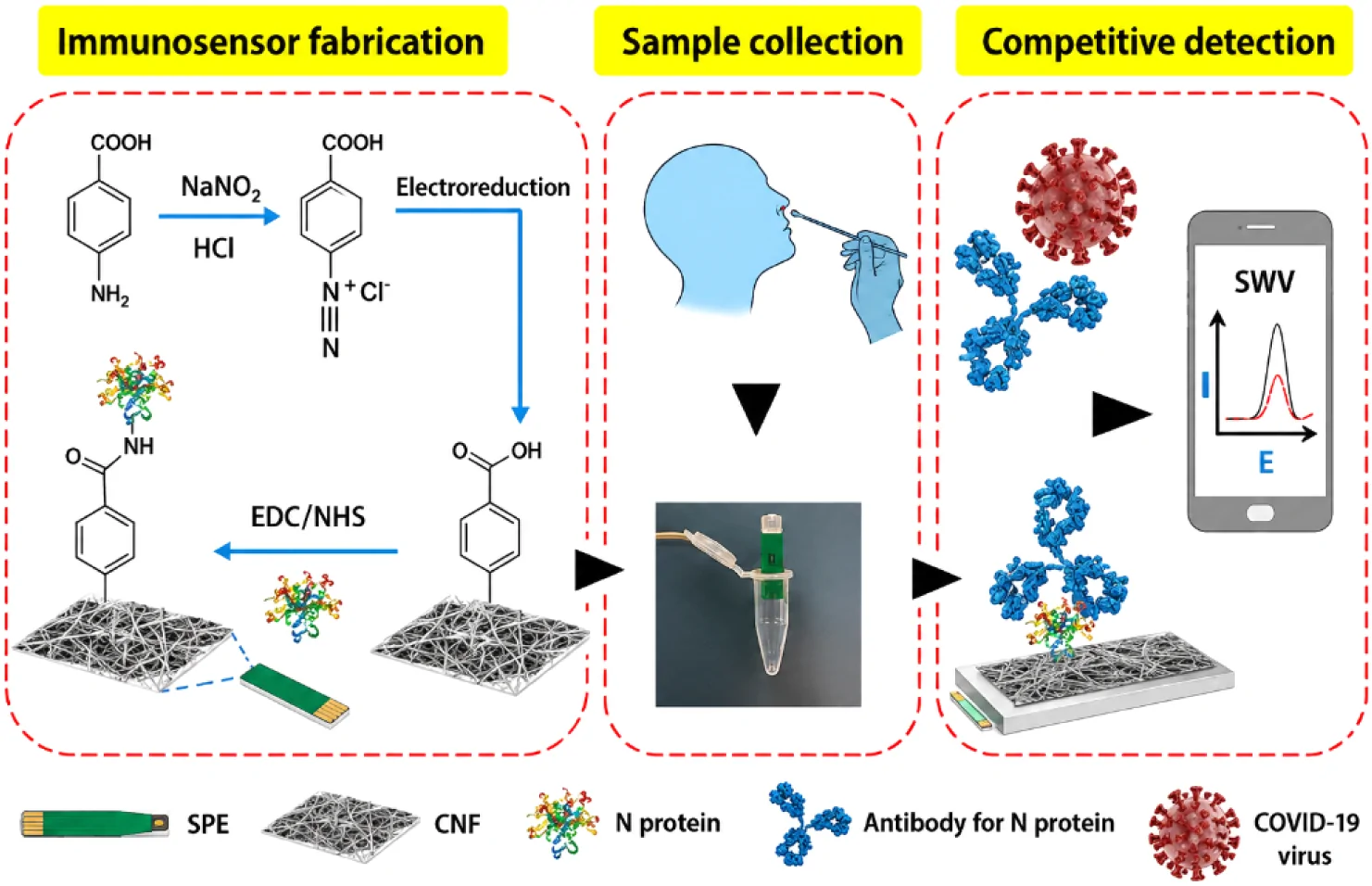
Schematic illustration of the fabrication and working principle of the paper-based electrochemical immunosensor for SARS-CoV-2 antibody detection. The device consists of a screen-printed carbon working electrode modified with graphene to enhance conductivity and surface area. Recombinant SARS-CoV-2 spike protein is immobilized on the electrode surface and serves as the biorecognition element to capture specific anti-SARS-CoV-2 antibodies present in the sample. Following antigen–antibody binding, a secondary enzyme-labeled antibody is introduced to form a sandwich immunocomplex. Upon addition of the enzymatic substrate, an electroactive product is generated, producing a measurable electrochemical signal proportional to the concentration of SARS-CoV-2 antibodies. The integrated paper-based platform enables sample introduction, immunoreaction, and electrochemical readout in a portable and rapid testing format. Adapted from *Analytical Chemistry*, 2021, 93, 1826–1833.^71^ Adapted with permission from the 2021 American Chemical Society.

##### Voltametric Electrochemical Immunosensors

Eissa *et al.* ^73^ reported on a label-free electrochemical biosensor for SARS-CoV-2 (N-protein) detection in nasopharyngeal samples. The biosensor platform is based on AuNPs that are functionalized with 11-mercaptoundecanoic acid (MUA) for attachment of an anti-SARS-CoV-2 nucleocapsid antibody. Their detection was achieved by monitoring the change in the electrochemical signal once the immunosensor was bound with the virus (N-protein). Thus, they observed a change in the reduction peak current. Their electrochemical immunosensor had a good sensitivity owing to the fast electron transfer rate and high surface area of the AuNPs. Moreover, they obtained a wide linear range of 1.0 pg/mL – 100 ng/mL and a LOD of 0.4 pg/mL. Moreover, they showed that their immunosensor had a high selectivity as it did not show significant responses with other viruses such as HCoV, MERS-CoV, Flu A, and Flu B. However, they had mentioned that there would be a possibility of cross-reactivity with SARS-CoV due to their similar structure. Furthermore, they tested the applicability of their immunosensor by using it for the detection of SARS-CoV-2 in clinical samples, and they found that their results were in good correlation with those of RT-PCR results. Thus, they concluded that their SARS-CoV-2 immunosensor was rapid, selective, sensitive, and low-cost, which is capable of being integrated into a handheld device and can be controlled via mobiles for point-of-care testing. Additionally, they were unable to validate what the best antigen would be for the detection of SARS-CoV-2 and have thus left the door open for further research. Wang *et al* ^74^ developed an immunosensor for the detection of the SARS-CoV-2 RBD S1. The sensor was based on an electrode platform that was modified with reduced graphene oxide Ag nanocomposite and incorporated an interaction system of biotin–streptavidin to link their synthesized nanocomposite and the RBP antibody that was tagged with biotin. This system provided the biotin-modified capture probes to be more efficiently immobilized and to improve the overall performance of the sensor. Due to the RBP antibody having an impaired electron transfer ability, they observed a decrease in redox peak currents. They made use of DPV for the detection of their target analyte, and the results showed that they obtained a linear range of 0.015 – 158.5 pg/mL and LOD of 7.2 fg/mL. They also show that their sensor’s LOD value and linear range are quite comparable to the other research work, and they provided justification as to why such is due to the properties of their nanocomposite and the biotin–streptavidin system. They evaluated the stability of their sensor through 20 continuous CV scans and obtained a current response that was only reduced by 6.4%. Their sensor was then stored at 4 °C for 16 days, where they tested the current response every 4 days and found that after 16 days, the initial response had only decreased by less than 10%. For the variation of the sensor, five identical sensors were tested, and the RSD value they obtained was 6.1%. Their proposed sensor showed good selectivity toward the RBD protein, as they observed no cross-reactivity with the interfering substance they chose, which was HPV, VZV, and SARS-CoV proteins. Lastly, the applicability of the immunosensor was tested by performing detection in artificial saliva samples, where they obtained recovery rates of 94.69 – 98.71%, showing the good stability and reproducibility of their immunosensor. Yadav *et al* ^75^ reported on an immunosensor for the detection of SARS-CoV-2 spike RBD protein in human serum samples. They made use of a glassy carbon electrode and modified it with a conductive and biocompatible biopolymer (chitosan) that was functionalized with molybdenum disulfide-graphene oxide (MoS_2_/rGO) nanohybrid. This functionalization led to a larger surface area, increased conductivity, and several other advantages. By making use of cross-linking chemistry (amide bond formation), their immunosensor showed a high affinity toward the spike RBD protein. They were able to confirm the formation of a biolayer through the binding of the recombinant spike protein to their synthesized nanohybrid by observing a decrease in the current response. They further confirmed this by analyzing their EIS results. Their as-synthesized material showed good stability since, after multiple scans, their observed peak remained almost unchanged. They used DPV as the electrochemical method for the detection of SARS-CoV-2 antibodies. Upon analysis, their immunosensor gave a wide range of detection from 10 ag/mL – 100 ng/mL and a LOD of 1.87 ag/mL. Their immunosensor showed negligible change in its peak current response in the presence of 5-fold interfering substances (dopamine, hemoglobin, transferrin, human serum albumin, and human immunoglobin G), highlighting the selectivity of their sensor. The reproducibility of their sensor had an RSD of 1 – 2%, which was obtained by measuring the SARS-CoV-2 antibodies on five modified electrodes in parallel in triplicate. Moreover, the stability was evaluated by storing the immunosensor at 4 °C for 14 days, and revealed that the peak current response only decreased by 92.90%. Lastly, they were able to successfully show that their immunosensor can differentiate between negative and positive samples.

##### Impedimetric Electrochemical Immunosensors

Mehmandoust *et al.* ^76^ reported on a novel label-free electroimmunosenor based on solvothermal-synthesized SiO_2_@UiO-66 core-shell nanocomposite for the detection of SARS-CoV-2 S protein, which is based on attaching the coronavirus to the ACE2 surface, which causes the virus to enter the organ. The results showed a wide dynamic range of 100 fg/mL – 10 ng/mL with a low limit detection of 100 fg/mL. They compared their results to previously published work that found that theirs were superior in terms of their low detection limit with low immunological response time (5.0 min), high selectivity in nasal samples, low toxicity of their nanocomposite, and reduced waste. Therefore, they developed a highly sensitive, relatively low-cost electro-immunosensor. They tested the selectivity of their immunosensor by studying the influence of interfering agents (Flu A, HCoV, l-glucose, l-cysteine, l-arginine, uric acid, dopamine, ascorbic acid, vitamin D, ribavirin, zanamivir, favipiravir, remdesivir, and tenofovir) and found no significant interference effects. Additionally, they calculated the relative error values to be less than 9%, showing that their immunosensor provides great selectivity toward SARS-CoV-2 S-protein. Furthermore, they detected the S-protein in a real sample (nasal) via their proposed immunosensor. They obtained satisfactory recovery values that indicated that the immunosensor was acceptable for the determination of SARS-CoV-2 S-protein in real samples. They likewise examined two samples in the nasal solution, one with the antigen and one without, and compared them with a PCR test. In the absence of the antigen, there was no electrochemical change for the immunosensor, and in the presence of the antigen, the immunosensor showed the same results as the PCR test, thus confirming that their developed immunosensor is reliable. Braz *et al* ^77^ developed an electrochemical immunosensor for the detection of SARS-CoV-2 anti-S antibodies. Their immunosensor was developed on SPCE and was based on AuNPs and synthetic peptides. These peptides were used for the binding of the AuNPs as well as a recognition site for their target analyte. Moreover, they observed that the Au binding peptide resulted in an enhancement of the analytical performance of the immunosensor. They made use of silico B-cell epitope prediction for the selection of the peptide that is specific to the S protein. They recognized that there were four possible regions (two of RBD and Monomer–Monomer Interaction) for the antibody recognition; however, based on their analysis, they selected a region between the 797 and 835 amino acid positions. Then, for the optimization of the peptide, they did spot synthesis of the selected region. They furthermore showed that the binding of the AuNPs and the peptides was superior to the peptides alone because these peptides proved to be unstable on the surface of the electrode, as there was a significant difference in the probe signal after a few washes. They made note that even though there is past literature on Au-binding peptides, they were the first to report on their use for SARS-CoV-2. They used DPV to evaluate the performance of their immunosensor, which achieved a LOD of 75 ng/mL, a sensitivity of 1.059 Μa/dec^,^ and a linear range of 75 ng/mL – 15 μg/mL. The selectivity of their sensor was tested in the presence of other antibodies, namely yellow fever, N protein, and a mixture of the yellow fever S and N proteins. The selectivity studies showed no significant changes in the presence of the antibodies on their own; however, the mixture of them did show a somewhat of impact, and the immunosensor remained highly selective toward the S-protein. Lastly, they analyzed positive and negative serum samples; they observed that there was no interaction of the nonspecific species of the negative serum sample, as there was no peak difference. However, for the positive serum sample, there was a clear difference (33%) in the signal response, but the immunosensor still showed sensitivity as it was able to differentiate between these positive and negative serum samples.

This reported research highlights the significance of immunosensors in SARS-CoV-2 diagnostics. This is further shown in Table 3, which shows a summary of the analytical performance and sensor modifications for SARS-CoV-2. The table likewise highlights the extensive research that has been reported for SARS-CoV-2 sensitive immunosensors. Despite this, most of the sensors/ELISA systems that are currently being developed rely on antibodies, which are the main components of the immune system’s recognition system.^78^ Although antibodies are commonly used as a routine part of the development process, they have several drawbacks, such as their large, complex structure and size, which can cause various defects.^79–81^ Additionally, one of the main drawbacks of using polyclonal antibodies is their variability in batch-to-batch conditions. This can prevent the development of protein engineering.^82^ Besides these technical issues, the use of antibodies can also lead to issues with cross-reactivity.^83^ This is because they are produced using animals. These issues raise ethical concerns as well as the associated costs.

**Table 3:** Representing Various Electrochemical Biosensors for Different SARS-CoV-2 Biomarkers^a^

Biosensor device	Biomarker	Active analytical layer	EC technique	LOD	Linear range	Advantages	Limitations	Ref.
Aptasensor								
**Electrochemical aptasensor**	S1	SPCE||Cu(OH)_2_ NRs|aptamer	SWV	0.03 fg/mL	0.1 fg/mL – 1.2 μg/mL	Total assay time is around 15 min, enabling fast turnaround suitable for point-of-care applications.	Tested only in saliva and VTM.	^52^
						Proven effective in saliva and viral transport medium; data aligned well with standard RT-PCR results for clinical specimens.	Detailed data on long-term aptamer shelf life, storage conditions, and reusability were not elaborated on and require further exploration.	
**Aptasensor**	NP	MEA chip||aptamer	EIS	7.0 pM	0.01 – 64 nM	Integrates microfluidics to concentrate analytes, enabling full detection within 15 s, far faster than traditional methods.	Though microfluidic enrichment accelerates detection, it adds hardware complexity, which may hinder portability or field deployment.	^56^
						Built on a low-cost MEA chip, which supports mass manufacturing and has potential for wide deployment.		
						Validated in varied matrices such as water, saliva, serum, and plasma, demonstrating its robustness across real-world samples.		
**Electrochemical aptasensor**	RBD	MPSF||LEGE|MB|aptamer	SWV	0.36 ng/mL	0.5 – 250 ng/mL	Leverages MPSF as both an MB carrier and bio-gate, integrated on LEGE, enhancing sensitivity and bringing low-cost, scalable fabrication potential.	Performance beyond saliva (e.g., nasal swabs, blood, wastewater) remains unexplored, limiting claims for universal applicability.	^53^
						Employs MB trapped in MPSF as an internal redox probe; MB is released upon aptamer–RBD binding, triggering a measurable current drop without additional labels.	Multi-component assembly, MPSF growth, MB loading, aptamer capping, and LEGE integration, which may introduce variability and raise manufacturing reproducibility issues.	
							Tested in spiked saliva only.	
**Electrochemical aptasensor**	RBD S1	SPCE||AuNP|streptavidin|aptamer	DPV	2.63 ng/mL	10 – 50 ng/mL	Maintains reliable performance in spiked saliva and ion-rich conditions, with excellent recoveries.	Total incubation (40 min aptamer +60 min analyte) results in ∼100 min assay duration, which is somewhat long for rapid point-of-care needs.	^55^
						Successfully tested on 32 nasopharyngeal swab specimens (3 positive, 29 negative), confirming real-world diagnostic potential.	Does not include stability studies; it’s unclear how long the biosensor remains reliable during storage.	
**Electrochemical aptasensor**	NP	SPCE||MSA-WTe_3_-QD|aptamer	CC	0.08 pg/mL	1 – 9 pg/mL	Validated in simulated real samples such as human serum, plasma, and urine, delivering recovery rates between 95% and 103%.	Sensor assembly requires controlled immobilization of QDs and DNA aptamer via EDC/NHS chemistry on SPCEs, which may challenge consistency and scale-up.	^61^
						Employs mercaptosuccinic-acid-capped WTe_3_ quantum dots to boost the electrochemical signal, enhancing sensitivity and signal stability.	Uses chronocoulometry, typically requiring bench-top or portable potentiostats, which are not readily available in low-resource or point-of-care settings.	
							The paper does not report shelf-life, storage stability, or the durability of the QD-modified sensors over time.	
**Fluorescence aptasensor**	SARS-CoV-2 virus	AuE||aptamer	EIS	5.1 fg/mL	2 pg/mL – 20 ng/mL	Incorporates both a FOEW and an EIS sensor using the same aptamer, increasing flexibility to match application needs.	FOEW, while fast, is less sensitive than EIS; EIS, although more sensitive, takes longer (up to 30 min).	^58^
						FOEW delivers results in ∼6 min.	FOEW requires optical fiber setups.	
						Uses aptamers recognizing chemically inactivated SARS-CoV-2, removing the need for live-virus handling.		
						EIS requires only a quick 10 min incubation (plus measurement) and can be processed within ∼15 – 30 min.		
**Aptasensor**	S1	Nanopipettes||APTES|aptamer	ICR	73.204 pg/mL	10^2^ −10^6^ pg/mL	Utilizes changes in surface charge at the nanopipette tip to transduce aptamer binding via modulation of ICR, eliminating the need for labeling or redox reporters.	Requires precise nanopipette pulling plus multi-step salinization and aptamer immobilization complex to standardize and scale.	^62^
						Ion-current measurements are on the scale of seconds, and nanopipettes are inexpensive to fabricate, reducing assay cost.	Nanopipettes (especially <50 nm tips) are mechanically fragile and prone to blockages, which may impair robustness during repeated use.	
							Stability tested over only 7 days; reusability over multiple uses wasn’t evaluated.	
Genosensor								
**Electrochemical genosensor**	RdRP	AuSPE||maleimide MP|strep-HRP20|cDNA|	CA	807 fM	1 – 60 pM	Uses maleimide-coated magnetic beads for immobilization, enabling efficient washing, separation, and concentration of target nucleic acids.	Requires multiple steps such as magnetic capture, target hybridization, secondary probe binding, enzyme labeling, and measurement, adding time and procedural complexity.	^63^
						Incorporates streptavidin-HRP (including poly-HRP20/80 complexes) for a strong electrochemical readout.	Reliance on streptavidin-HRPraises issues around enzymatic stability, activity degradation, and cold-chain shipment requirements.	
						Successfully tested on RNA from SARS-CoV-2 cell cultures and in spiked matrices, demonstrating early-stage applicability near the point of care.		
**Electrochemical genosensor**	RdRP	paper||GNC|Au@CD NPs|cDNA	DPV	0.15 pM	0.50 pM – 12 nM	Employs toluidine blue intercalation, resulting in stronger binding to the cDNA-RdRP duplex, yielding a clear voltammetric signal without enzymatic amplification.	Electrode modification is a multi-step process (GNC deposition, nanoparticle conjugation, DNA immobilization) and is relatively slow.	^64^
						Shows low variability (RSD ∼2.2%) thanks to strong GNC adhesion and stable Au@carbon-dot/DNA bioconjugates.	Relies on a redox indicator (toluidine blue), which may require chemical handling/preparation incompatible with low-resource POC environments.	
**Electrochemical genosensor**	cDNA and creatine	G-PLA||AuPs|cDNA	EIS	0.30 μmol/L	0.050 – 3.2 mmol/L	Total hybridization time is 30 min, which is relatively swift for nucleic acid assays based on hybridization.	Though non-enzymatic, the sensor relies on hybridization-induced current drop without amplification, limiting sensitivity enhancement.	^66^
						The platform also tested successfully for creatinine detection, demonstrating versatility for viral and biomarker sensing.	Sensor detects cDNA without preceding reverse transcription; applicability to direct viral RNA detection remains untested.	
**Electrochemical genosensor**	RdRP	SPCE||GONC|DNA probe	EIS	186 × 10^–9^ M	10^–10^ – 10^–5^ M	Offers a quick assay time of about 30 min, facilitating timely results essential for effective diagnostics.	The biosensor’s ability to distinguish between SARS-CoV-2 and other similar pathogens needs further validation to ensure accurate diagnosis.	^67^
						Utilizes graphene nanocolloids that serve both as a conductive platform and as a signal amplifier, enhancing the overall performance of the biosensor.	The consistency of the sensor’s performance across different batches and over time requires further assessment to ensure reliability.	
Immuno-sensor								
**Electrochemical immunosensor**	NP	SPE||CNF|Ab1	SWV	2.2 fmol/L	1 – 10,000 fmol/L	Combines sample collection and detection into a single device by coating screen-printed electrodes with cotton, simplifying the testing process.	The manual swabbing process may introduce variability, affecting the consistency of results.	^71^
						Eliminates the need for sample transfer or pretreatment, streamlining the testing process.		
**Electrochemical immunosensor**	NP	SPC||AuNPs|MUA|Ab	SWV	0.4 pg/mL	1.0 pg/mL – 100 ng/mL	Initial testing with clinical samples indicated good correlation with RT-PCR results, suggesting practical applicability.	The manual sample preparation process may introduce variability, affecting the consistency of results.	^73^
						The immunosensor operates without the need for additional labeling agents, simplifying the detection process.	The sensor is designed for detecting a single antigen, which may limit its ability to differentiate between different pathogens or variants.	
**Electrochemical immunosensor**	S1	SPCE||cyst-SiO_2_@UiO-66|Ab	DPV	100 fg/mL	100 fg/mL – 10 ng/mL	The sensor provides results within 5 min, facilitating timely diagnostics.	The manual sample preparation process may introduce variability, affecting the consistency of results.	^76^
						Initial testing with nasal samples indicated good recovery percentages, suggesting practical applicability.		
**Electrochemical immunosensor**	S1	GCE||PtPdRu NDs|Ab1|S1|SiO_2_NS-Ab2	CA	51.74 fg/mL	1.0 pg/mL – 1.0 μg/mL	Utilizes the peroxidase-like activity of PdPtRu nanozymes to amplify the electrochemical signal, enhancing detection sensitivity.	The preparation of PdPtRu trimetallic nanozymes involves complex synthesis procedures, which may limit scalability and increase production costs.	^72^
**Electrochemical immunosensor**	RBD S1	AuE||Ag-rGO-Nf|strep|Ab	DPV	7.2 fg/mL	0.015 – 158.5 pg/mL	Use of Ag-rGO nanocomposites provides a relatively low-cost material platform compared to some noble metals or complex nanomaterials.	Although selectivity was tested, real complex biological fluids like serum or saliva may introduce interference that affects sensor performance and needs further validation.	^75^
**Electrochemical immunosensor**	RBD S1	GCE||CS–MoS_2_|rGO|Ab	DPV	2.38 zg/mL	10 zg/mL −100 ng/mL	The sensor has been successfully tested with real biological samples, demonstrating practical applicability and reliability in complex matrices.	The preparation of the MoS_2_-rGO nanohybrid and functionalization steps involve multiple stages that require careful control, which could challenge reproducibility and scalability.	^75^
**Electrochemical immunosensor**	S1	SPCE||Pept|AuNP|Ab	DPV	30 ng/mL	75 ng/mL – 15 μg/mL	Peptide synthesis and immobilization onto gold electrodes are relatively low-cost and straightforward, facilitating easier sensor production.	Long-term stability and resistance of the peptide layer under varying temperature, pH, and storage conditions require further study.	^77^
**Electrochemical immunosensor**	RBD S1	SPCE||AuNPs|cyst|IgY Ab	EIS	5.65 pg/mL	11.56 – 740 ng/mL	IgY antibodies from chickens offer several benefits, such as cost-effective production, high yield, and ethical advantages compared to mammalian antibodies.	Complex biological fluids could interfere with detection, necessitating additional work to confirm robustness in diverse sample types.	^78^
							The long-term stability of the IgY antibodies on the sensor surface under varied storage conditions needs further investigation.	
**Electrochemical immunosensor**	S1	SPCE||AuNPs|cyst|Ab	SWV	36.3 pg/mL	250 pg/mL – 20 μg/mL	The immunosensor is designed to be disposable and uses environmentally friendly materials, reducing electronic waste and environmental impact.	Being disposable limits reusability, which, while environmentally conscious, may increase overall resource consumption if not managed sustainably.	^99^
						Requires a small volume of biological sample, which is advantageous for patient comfort and sample availability.	The stability of the disposable sensor over extended storage or varied environmental conditions requires further investigation.	
						The sensor uses inexpensive materials and simple fabrication processes, making it affordable for large-scale production and widespread use.		
**Electrochemical immunosensor**	S1	SPCE||GQDs@AuPt|Ab	EIS	60.14 ng/mL	0.05 – 1.5 μg/mL	The immunosensor enables direct detection of SARS-CoV-2 without the need for PCR amplification or labeling, simplifying the assay and reducing cost and time.	Although promising, more extensive testing with real clinical samples is needed to confirm practical applicability.	^100^
						Avoidance of PCR and labeling reduces costs and technical complexity, making the sensor more accessible for point-of-care or field testing.		
**Electrochemical immunosensor**	S1	GCE||N-HRGO|TH|Ab	EIS	0.3 pg/mL	1 pg/mL −10 ng/mL	The sensor maintains its performance over time, making it suitable for long-term use.	Using nitrogen-doped holey graphene decorated with thionine molecules adds complexity to the sensor’s production.	^101^
**Electrochemical immunosensor**	recombinant trimeric S1	ITO||ZnONR|Ab	EIS	19.34 ng/mL	200 – 1200 ng/mL	The synthesis and modification of ZnO nanorods are relatively low-cost and straightforward, favoring scalable production.	The ZnONR-modified fluorine-doped tin oxide substrates require specialized preparation.	^102^
						The platform allows for the fast detection of antibodies in serum samples from both convalescent and vaccinated individuals.		
**Electrochemical immunosensor**	S1	SPCE||Cu_2_O NC|ProtA|S protein Ab	EIS	0.04 fg/mL	0.25 fg/mL – 1 μg/mL	The sensor design allows label-free detection and involves straightforward electrode modification with Cu_2_O nanocubes, making the fabrication process relatively simple and cost-effective.	The performance in complex biological samples like serum or saliva may be affected by nonspecific adsorption or matrix interferences, which could require additional sample preparation.	^103^
						The sensor demonstrates acceptable stability and reproducibility over multiple uses and storage periods.		
**Electrochemical immunosensor**	NP	AuSPE||cAb|N protein|dAb-HRP	CA	50 pg/mL	0 – 10 ng/mL	The microfluidic design enables fast sample processing and measurement, significantly reducing total assay time compared to conventional methods.	The microfluidic and magnetic bead integration may require precise fabrication and handling, which could limit widespread use without specialized equipment.	^104^
						The microfluidic platform requires minimal sample volume, making it less invasive and more practical for clinical settings.	Despite improvements, matrix effects from serum components may still affect sensor accuracy, requiring optimized surface chemistry and blocking agents.	
						The compact and automated microfluidic setup allows for potential integration into portable diagnostic devices.	The complexity of the microfluidic system and magnetic bead reagents may increase costs and pose challenges for mass production.	
Nanobody-based biosensor								
**Photoelectrochemical immunosensor**	S1	ITO||Au@TiO_2_|Nb	PEC	5 fg/mL	0.015 – 15000 pg/mL	Nanobodies provide high affinity and specificity to the spike protein, with a smaller size facilitating better surface immobilization and improved sensor performance.	PEC detection requires a light source and a photoelectrochemical measurement setup, which may limit accessibility in low-resource settings.	^92^
						PEC detection typically offers low background signals, leading to improved signal-to-noise ratio and assay accuracy.	PEC sensors can be sensitive to environmental factors such as light intensity and temperature, which may affect reproducibility and require controlled conditions.	
**Whole-cell biosensor**	S1	YS||AuNPs|HRP-Ab	CA	0.037 μg/mL	0.01 – 1 μg/mL	Nanobody display on whole-cell biosensors provides particular binding to the SARS-CoV-2 target proteins due to the high affinity of nanobodies.	Use of genetically engineered whole cells may raise biosafety and regulatory challenges for clinical or field deployment.	^95^
						The use of a colorimetric readout allows for simple, rapid, and visible detection without the need for complex instrumentation, making it accessible for point-of-care or resource-limited settings.	Whole-cell biosensors can be sensitive to environmental factors such as temperature, pH, and nutrient conditions, potentially affecting stability and reproducibility.	
						This approach integrates biological recognition and signal transduction in a single living cell, which can amplify signals and improve detection sensitivity.	Colorimetric assays often provide qualitative or semi-quantitative results, which might be less precise than electrochemical or fluorescence-based methods.	
						Use of whole cells and nanobodies reduces reliance on expensive purified proteins or antibodies, lowering production and assay costs.		

a Copper hydroxide nanorods = Cu­(OH)_2_ NRs, mesoporous silica film = MPSF, laser engraved graphene electrode = LEGE, methylene blue =MB, aminopropyl-triethoxylsilane =APTES, mercaptosuccinic acid-capped tungsten telluride quantum dot = MSA-WTe_3_-QD, magnetic particles = MP, streptavidin-poly horseradish peroxidase 20 = strep-HRP20, graphite nanocrystals = GNCs, gold carbon dot nanoparticles = Au@CD NPs, G-PLA = graphene polylactic 3D-printed electrode, graphene oxide nanocolloids = GONC, carbon nanofibers = CNF, cyst = cysteamine, graphene quantum dots coexisting with gold and platinum nanoparticle nanocomposite = GQDs@AuPt, pristine holey graphene -thionine = N-HRGO|TH, silver reduced graphene oxide nanocomposite = Ag-rGO, zinc oxide nanorod = ZnONR, Cu_2_O nanocubes = Cu_2_O NCs, staphylococcal protein A = ProtA, cAb = capture antibody, dAb= detection antibody, YS = yeast surface, spike protein = S1, nucleus protein = NP, recombinant binding domain protein = RBD, 11-mercaptoundecanoic acid = MUA, nanodots = NDs, nanosphere = NS, nafion = Nf, conducting biopolymer functionalized molybdenum disulfide-reduced graphene oxide = CS–MoS_2_|rGO, gold-binding peptide = Pept|AuNP, indium tin oxide = ITO, screen printed carbon electrode = SPCE, glassy carbon electrode = GCE, photoelectrochemical = PEC, differential pulse voltammetry = DPV, square wave voltammetry = SWV, chronoamperometry = CA, chronocoulometric = CC, electrochemical impedance spectroscopy = EIS, ion current rectification = ICR, fiber-optic evanescent wave = FOEW, Viral Transport Medium = VTM.

#### Nanobody-Based Biosensors

Due to technological advancements in the field of biologics, there is a growing movement toward the use of recombinant antibodies and non-antibody binding proteins (NABPs). This method allows the continuous production of a stable protein with no variation. Moreover, the obstacles that occur when using conventional antibodies can be overcome by a single entity that has the full antigen-binding properties of a functional antibody; this is known as a nanobody (Nbs/V_HH_). These new products are designed to carry a variable domain of heavy-chain antibodies found in animals from the Camelidae family.^84^ Nanobodies are small fragments (15 kDa) that have a less complex structure with antigen-binding units that are more effective.^85^ Besides being useful in drug screening, nanobody technology can also be used to address the issue of the reproducibility crisis in the industrial and academic communities.^86^ Compared to conventional antibodies, nanobodies have various advantages, such as their high thermostability, improved solubility, flexibility, ease of expression, and ability to interact with a variety of microorganisms.^87^ V_HH_s have a shorter half-life, which allows the kidneys to quickly clear the unbound portion of the product. They can also be produced in large quantities using various types of bacteria and yeast.^84^ It is also cheaper to develop and maintain these types of antibodies than to produce multiple types of conventional antibodies. Through their low cost and high performance, Nbs have been able to be produced in bacterial cells. Due to the unique properties of these nanobody antibodies, they have been able to be used as recognition elements for various harmful substances and small molecules. They have been regarded as outstanding features in the field of therapeutic^88^ and diagnostic^89^ applications. The first reported application of these antibodies was in a chip for the use of a surface plasmon resonance device.^90^ However, Nbs have beneficial properties for developing biosensors, understanding biological processes, and generating innovative therapeutics for the treatment of diseases. Thus, Nbs might solve several problems encountered with classical antibodies to detect clinically relevant antigens with good sensitivity even under harsh conditions, creating many opportunities to diagnose diseases or infections. Multiple studies have shown that Nbs can neutralize the spike receptor binding domain of the SARS-CoV-2 antigen.^85^ Furthermore, the lack of a rapid and accurate method for the diagnosis of SARS-CoV-2 has hindered the progress of the investigations. Pagneux *et al* ^21^ used a nanobody-functionalized voltammetric sensor based on a poly­(ethylene)­glycol-modified gold electrode for the detection of the whole SARS-CoV-2 in both human saliva and nasopharyngeal swab samples. The nanobodies used were highly oriented and specific to the spike protein RBD. Their device was able to provide results in less than 10 min of exposure and showed high specificity to the target viral particles in the saliva and nasopharyngeal samples. The developed sensor obtained a LOD of 1.2 x 10^4^ viral RNA copies/mL and could distinguish between different human coronavirus strains and other respiratory viruses, with 90% negative and positive percentage agreements on 80 clinical samples, when compared to RT-qPCR for the nasopharyngeal samples. Moreover, they identified that the sensor was potentially falsified by the presence of saliva proteases and other saliva proteins. Additionally, they tested the long-term stability of their sensor and found a 4% loss when tested with RBD. Thus, their results showed that their device sensitivity was comparable to the RT-PCR method. They concluded that even though their device could be improved at that time, the tool was very efficient, rapid, portable, manageable, and could be used directly in emergency/consulting rooms. Ruiz-Vega *et al* ^91^ used a label-free silicon photonic nano biosensor based on bimodal waveguide (BiMW) interferometric technology that was functionalized with nanobodies for targeting the SARS-CoV-2 RBD. They found that their nanophotonic biosensor was able to detect the viral particles in less than 20 min. Moreover, their approach relied on the direct capture of the viral particles, and the optimized methodology allowed for the detection of the SARS-CoV-2 virus with outstanding sensitivity, below 200 TCID50 per mL, being able to provide accurate viral load determination within a broad dynamic range. Due to their short assay completion time, it showed that their biosensor could eventually be used in decentralized systems in healthcare facilities. Additionally, they used the modified electrode with both conventional monoclonal antibodies and the bioengineered nanobodies for the detection of the SARS-CoV-2 RBD. The LOD and LOQ values obtained for the bioengineered nanobodies were 162 FFU/mL and 727 FFU/mL, respectively. These values were 3 times greater than those obtained for the monoclonal antibodies; thus, they showed that their specific modified biosensor has excellent affinity for the RBD and can enhance the performance of the sensor. Based on the results that they obtained, they further investigated the direct capture, detection, and quantification of real SARS-CoV-2 viral particles employing bioengineered Nb as recognition elements and obtained outstanding LOD and LOQ values, indicating that they could directly visualize and quantify in real-time. They then also assessed the sensor toward the detection of the whole SARS-CoV-2 virus in a common viral transport medium; they obtained LOD and LOQ values that were slightly greater than their originally obtained values (purified samples); however, they were still lower than the clinical cutoff. They concluded that their novel method has the potential to be implemented in healthcare and clinical practices. Chen *et al* ^92^ developed a label-free photoelectrochemical immunosensor based on a nanobody that was immobilized onto a surface that was modified with Au-deposited TiO_2_ nanomaterials for the detection of the SARS-CoV-2 spike protein. The schematic diagram of the fabrication of the nanobody-based biosensor is displayed in Figure 3. They showed that their proposed immunosensor had satisfactory sensitivity, stability, reproducibility, and applicability. They were able to base those conclusions on the following results that they obtained. Their immunosensor showed a linear detection range from 0.015 – 15000 pg/mL, and a LOD as low as 5 fg/mL. They also compared their method to previously reported methods for the detection of the SARS-CoV-2 spike protein. Although they found that some of the methods (opto-microfluidic chip, phononic, and field-effect transistor sensors) had fast analysis and high reliability, they were expensive in terms of running and fabrication costs. Moreover, they found that their method proved to be sensitive, cost-effective, and rapid. They performed selectivity tests of their immunosensor by detection of the SARS-CoV-2 S protein against several interfering substances, and they found no significant effects on the photocurrent response. The stability and reproducibility were likewise tested, and they found that when compared to the standard photocurrent value, the RSD was below 4%, showing its high reproducibility. Lastly, they detected SARS-CoV-2 S protein in oropharyngeal swab samples, and the recovery with S protein was calculated to be 99.83%, and the RSD value was 1.04%, showing the immunosensor’s great applicability. However, they were unable to perform real-time monitoring of real samples. Thus, for future work, they stated that they would like to collaborate with local hospitals. Guo *et al* ^93^ used nanobody-functionalized organic electrochemical transistors with a modular architecture for the rapid analysis of specific SARS-CoV-2 antigens in complex bodily fluids. The nanosensors combine a solution-processable conjugated polymer and nanobody-SpyCatcher fusion proteins on disposable gate electrodes by organic electrochemical transistors (OECT) signals. This nanobody is promising to be extended as a bioreceptor to detect SARS-CoV-2 antigen if the corresponding specific nanobody is available to conjugate with the NIR fluorescent nanomaterials. Nanobodies normally can target SARS-CoV-2 infections at ultra-low doses, which will show advantages in reducing the biohazard release from the sampling process. Ma *et al* ^94^ developed a nanoplasmonic biosensor for the quantification of the SARS-CoV-2 RBD S1 in serum. They immobilized a mixture of nanobodies (NB1D6 and NB4E9) on an AuNP fluorine-doped tin oxide platform for the detection of the target protein. By mixing the nanobodies, they found that they achieved a higher detection sensitivity when compared to any nanobody on its own. Furthermore, they made use of a homemade 36-well plate for simultaneously functionalizing 36 biosensor chips. Their sensor was based on a sandwich type with the primary antibody tagged with His and the secondary antibody tagged with DYDDDDK; however, both were conjugated with CoraLite 488. They analyzed their immunofluorescence results to confirm that the nanobodies successfully formed a self-assembly layer on the modified platform. The two nanobody-based biosensors gave a low LOD of 0.01 ng/mL. Their sensor likewise showed good specificity and selectivity. They obtained those results by using their biosensor for not only detecting their target analyte but also inferring protein solutions such as PBS, BSA, AFP, CEA, IgG, and CA15-3, and they found that these interfering substances showed a negligible absorbance on the substrate. They noted that even though the mixture of their nanobodies had an improvement in the detection response, its sensitivity and selectivity were still the same as a single nanobody. Wang and co-workers^95^ reported on the use of biosensor strips based on laser-scribed graphene (LSG) for the detection of COVID-19. They used direct laser scribing on a polyimide substrate to make the LSG. This fabrication was not only cost and time-efficient but also very simple and allowed for mass production. Furthermore, their system was based on the use of an EsterLigase (a split-ester bond ligase) system for an RBD S1-specific nanobody to be immobilized onto their platform. This allowed for their probe to have a maintained out-of-plane orientation for the probe-target recognition process to be as efficient as possible. Typically, their system principles were based on a modified E-Tag peptide covalently linked to a graphene electrode after being immobilized by a C-terminal polyester bond. A spike-associated V_HH_ E nanobody, which was genetically fused with a ligase, was then captured and covalently linked to the electrode through a spontaneous catalytic ester bond between the E-TAG and the EsterLigase ligase. A buffer solution containing a ([Fe­(CN)_6_]^3‑/4–^) was then used to determine the presence of the SARS-CoV-2-RBD S1 via electrochemical studies (EIS). The sensor offered a linear detection range of 150 pM – 15 nM, a LOD of 7.68 pM, with a quick readout of about 30 min. They noted that their biosensor can also be utilized to identify other viral proteins. Similarly, He *et al* ^96^ reported on a whole-cell nanobody-based biosensor using colorimetric analysis for the determination of the SARS coronavirus spike protein. Their sensor was based on a genetically engineered yeast surface, which served as their bioreceptor and showed a high capture efficiency. They then used an AuNP-HRP nanobody-bioconjugate as their signal transducer to increase the sensor’s detection sensitivity. Their sensor could detect the SARS-CoV-2 S1 in a range of 0.01 – 1 μg/mL, with a LOD of 0.037 μg/mL. The major findings in their research were: (i) the use of engineered yeasts as bioreceptors ensures that the nanobodies can capture the affinity of the whole cell; (ii) the reporter AuNPs can improve the sensitivity and specificity of the assay; and (iii) the developed whole-cell biosensor can be used to detect different variants of the SARS-CoV-2, which is a promising application in the high-throughput analysis of this virus.

**Figure 3:**
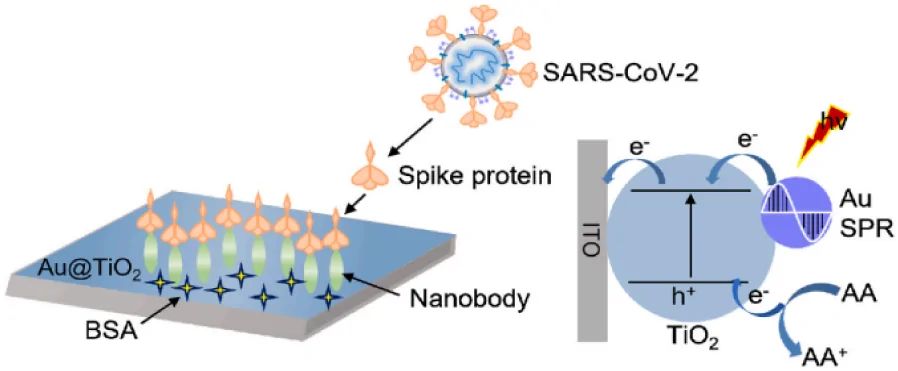
Schematic illustration of the nanobody-based photoelectrochemical (PEC) biosensor for SARS-CoV-2 spike protein detection. Nanobodies specific to the SARS-CoV-2 spike protein are immobilized onto an Au@TiO_2_ photoactive electrode, while bovine serum albumin (BSA) is used to block nonspecific binding sites and improve assay selectivity. Upon exposure to the sample, the target spike protein binds specifically to the immobilized nanobodies, forming an antigen–nanobody complex on the electrode surface. Under light irradiation (hν), TiO_2_ generates electron–hole pairs, while the gold nanoparticles enhance light harvesting through localized surface plasmon resonance (SPR). The photogenerated electrons are transferred through the electrode to produce a photocurrent, whereas the holes are scavenged by ascorbic acid (AA), which is oxidized to AA^+^. Binding of the spike protein alters interfacial charge-transfer processes and modulates the PEC response, enabling quantitative detection of SARS-CoV-2. Adapted from *Analytica Chimica Acta*, 2022, 1211, 339904.^92^ With copyright permission from Elsevier B.V.

Based on Table 3, the nanobody-based biosensors for the detection of SARS-CoV-2 are comparable to the analytical performance of biosensors. However, it is evident that to date, there are not many published articles relating to the research of nanobody-based biosensors for this specific disease. Nonetheless, previous research on these types of biosensors for other diseases provides proof of concept that nanobodies do indeed have enhanced chemical properties.^97^ This highlights the need for more intensive and extensive research into this area of biosensors.^98^

### Electrochemiluminescence-Based Electrochemical Biosensors

The increasing need for enhanced sensitivity, stability, and selectivity has shifted from electrochemical biosensors to ECL biosensors. ECL biosensors are ideal for various bioanalytical applications, such as those used in pharmaceutical research and clinical diagnostics. Conventional electrochemical biosensors rely on signals from the redox reactions at the electrode surface. Unfortunately, these signals can be affected by background noise generated by interfering substances. On the other hand, biosensors that utilize ECL technology generate light through a luminescent reaction, which eliminates the interference.^105^ The advantages of ECL biosensors over their electrochemical counterparts are their stable and reproducible nature. Unlike electrochemical biosensors, which can deteriorate over time, ECL systems and reagents are generally stable and can perform consistently. This ensures that they are ideal for high-volume automated systems, like those used in clinical diagnostic applications. Thus, ECL biosensors have lower detection limits, which makes them ideal for detecting traces of biomolecules in environmental and clinical samples. An ECL biosensor can achieve high selectivity due to its use of co-reactants and luminophores. This makes them ideal for drug discovery and medical diagnostics. In addition to reducing false positives, this technology also boosts the specificity of analytes. ECL biosensors feature an extensive dynamic range, which enables the simultaneous detection of elevated and low analytes with little compromise in accuracy.^106^ This versatile feature is particularly advantageous in applications like SARS-CoV-2 detection, where precise determination is required. Due to the increasing demand for efficient and accurate biosensing technologies, ECL is becoming a promising platform for the development of next-generation systems.

Tamara Guerrero-Esteban and co-workers^107^ discuss the development of a novel method for detecting SARS-CoV-2 in wastewater. Their method utilized a carbon nanodot-amplified ECL immunosensor to detect the SARS-CoV-2 S1. The carbon nanodots served a dual purpose: they amplify the ECL signal and provide functional groups for covalent interaction with the SARS-CoV-2 Spike S1 antibody. Their proposed ECL immunosensor demonstrated high specificity and sensitivity, with a detection limit of 1.2 pg/mL. It showed excellent stability and can detect SARS-CoV-2 in both river and urban wastewater. They highlighted that their method offers a reliable and efficient platform for early virus detection, which can be used to monitor the circulation of the virus in the population and detect potential resurgences of COVID-19. Similarly, Hosseini *et al* ^108^ reported on a highly sensitive and high-throughput sandwich ECL immunosensor for the detection of the SARS-CoV-2 spike protein. This ECL immunosensor utilizes AuNPs coated with BSA and luminol nanocomposites to enhance the ECL signal. The developed immunosensor demonstrates a linear detection range from 10 ng/mL to 10 μg/mL and a LOD of 1.93 ng/mL. It offers rapid analysis within 90 min and is easy to use, making it suitable for early clinical diagnosis of COVID-19. The sensor’s excellent recovery in detecting SARS-CoV-2 highlights its potential for widespread diagnostic applications. Then, Yu Wen and co-workers^109^ reported on a biosensor that utilized morphology-controlled iridium complex nanomaterials to detect the SARS-CoV-2 nucleocapsid protein with high sensitivity and specificity. The iridium complex nanomaterials are designed to enhance the ECL signal, providing a robust platform for the detection of the N protein. The biosensor demonstrates excellent performance, with a low detection limit (47 fg/mL) and a wide linear range, making it suitable for early diagnosis and monitoring of COVID-19. The study highlights the potential of this ECL biosensor as a powerful tool for rapid and accurate detection of SARS-CoV-2 in clinical samples. The analytical performance of an ECL-based biosensor was likewise studied by Villa-Manso *et al*,^110^ who reported on the development of a novel immunosensor based on electrochemiluminescence resonance energy transfer (ECL-RET) for the sensitive detection of the SARS-CoV-2 N protein. This immunosensor utilized bifunctional core@shell nanoparticles composed of a platinum-coated gold core, decorated with small gold inlays (Au@Pt/AuNPs), which acted as ECL acceptors. Their immunosensor demonstrated a wide linear response to the concentration of the N protein, with a detection limit of 1.27 pg/mL. It exhibited high stability and specificity, showing no response to other proteins related to different viruses. The immunosensor was successfully used to quantify the N protein in saliva samples, with results consistent with those provided by a commercial colorimetric ELISA kit. These reported developments on ECL-based biosensors offered a reliable tool for early and effective detection of SARS-CoV-2, contributing to the protection of public health. Table 4 summarizes the analytical performance and information about these reported ECL biosensors for SARS-CoV-2. Thus, when Table 4 is compared to Table 3, it is evident that there is a prominence of research on electrochemical biosensors for SARS-CoV-2 detection over ECL biosensors. This can be attributed to several factors, the main one being that the technology behind electrochemical biosensors is well-established, with extensive prior research and development, allowing for quicker adaptation to detect SARS-CoV-2. However, it is crucial to shift our focus to ECL biosensors due to their unique advantages mentioned earlier. By investing more research into ECL biosensors, we can develop advanced diagnostic tools that complement existing electrochemical methods and enhance our overall response to infectious diseases.

**Table 4:** SARS-CoV-2 Sensitive ECL-Based Immunosensors^a^

Biomarker/generic material	Active layer	Co-reactant, Luminophore	Linear range (ng/mL)	LOD (ng/mL)	Ref.
**S1**	SPCE||CNDs|S1-Ab	CNDs, [Ru(bpy)_3_]^2+^	0.0025 – 0.24	1.2 × 10^–3^	^107^
**S1**	GCE||Au|Ab1|S1|luminol-Au@BSA-Ab2	H_2_O_2_, luminol	10 – 10 × 10^3^	1.93	^108^
**NP**	GCE||Ir NPs|Ab1|NP|Ab2@Au NPs-NA	NA-Au NPs, Ir(ppy)_3_	1 × 10^–4^ – 10	4.7 × 10^–4^	^109^
**NP**	AuSPE||cAb-MB|NP|dAb- Au@Pt-AuNPs	TprA, [Ru(bpy)_3_]^2+^	0.0025 – 0.24	1.27 × 10^–3^	^110^

a Carbon nanodots = CNDs, iridium nanoparticles = Ir NPs, spike protein = S1, nucleocapsid protein = NP, noradrenaline = NA, electrochemiluminescence = ECL, resonance energy transfer = RET, capture antibody = cAb, detection antibody = dAb, gold nanoparticles = AuNPs, platinum = Pt, screen printed carbon electrode = SPCE, glassy carbon electrode = GCE, nitrogen-doped carbon nanodots = CNDs, noradrenaline = NA, tris­(2-phenylpyridine)­iridium­(III) = Ir­(ppy)_3_, (tris­(bipyridine)­ruthenium­(II)) = [Ru­(bpy)_3_]^2+^, Tripropylamine = TprA and Antibody = Ab.

### Multiplex/Simultaneous Detection of SARS-CoV-2

Multiplex detection is the detection of multiple analytes, in this case, biomarkers, in one single run. The combination of several biomarkers in the same test improves the overall specificity, sensitivity, efficiency, and accuracy of the detection. Additionally, it allows for the disease outcome to be predicted when various antibody signatures are identified and permits the immunological response to be assessed impartially for future vaccines. Likewise, multiplex detection allows for the generation of false-negative results to be avoided. Thus, based on the benefits of multiplex detection, there has already been a wide range of research that investigated the simultaneous detection of SARS-CoV-2 biomarkers. Table 5 highlights the capability of multiplex and nanomaterial-enhanced biosensors to simultaneously detect multiple SARS-CoV-2 biomarkers with broad dynamic ranges and markedly improved sensitivity compared to single-analyte assays.

In the study by Wang *et al*,^111^ they fabricated a dual-mode LFIA based on magnetic quantum dots with triple quantum shells (MagTQD) for the simultaneous detection of both the SARS-CoV-2 nucleocapsid protein and spike antigens. Under direct and enriched modes, the LOD values of the N and S protein antigens were 0.5 and 1 pg/mL, respectively. They further examined the specificity, stability, and accuracy of the assay in real biological specimens and showed that their proposed method successfully detected the antigens in the biological samples with a rapid detection speed (10 – 35 min), flexibility, good reproducibility (CV < 12.3%), good specificity and stability, and high sensitivity (0.5 – 1 pg/mL). Bayin *et al* ^112^ developed an LFI assay based on a giant magnetoresistance (GMR) sensing system for the simultaneous detection of anti-SARS-CoV-2 IgM and IgG. The sensing platform was likewise modified with superparamagnetic nanoparticles (SMNPs) as the sensing probe to analyze the magnetic intensity of the target analyte. Thus, most magnetic signals captured by the test strip will be able to be detected using their developed SMNPs-based LFIA. Their system was based on a sandwich type, whereby on the nitrocellulose (NC) membrane, the capture antibodies were immobilized. The NC membrane thus consisted of the two test lines: TL1 (anti-human IgG) and TL2 (anti-human IgM), and one control line (goat anti-rabbit IgG). The detecting antibodies (N protein-labeled SMNPs) were then coated on the glass fiber of the strip, allowing the antibodies (IgG and IgM) to combine with the NP-SMNP, which resulted in a color band and magnetic signal appearing. Upon analyzing their results, they observed that they were able to obtain reliable results in 10 min with LOD values of 10 ng/mL and 5 ng/mL for IgM and IgG, respectively. They then also tested their test strip in serum samples; thus, they used the anti-SARS-CoV-2 IgG and IgM in fetal bovine serum solution, and upon magnetic and color intensity analysis, they observed that TL1 and TL2 still showed prominently visible bands. Furthermore, they showed that their developed GMR platform was able to be adapted to be used as an intelligent phone-based Internet of Medical Things (IoMT) platform, allowing doctors easy access to the patient’s information (preliminary test results). Lin *et al* ^113^ established a microfluidic immunoassay that contained a fluorescence detection analyzer, multiplex immunoassays, and SARS-CoV-2 diagnostic microchips for the simultaneous detection of three SARS-CoV-2 biomarkers, namely IgG, IgM, and antigen. Their integrated method was able to successfully detect the antibodies and the antigen in pharyngeal swabs from 26 COVID-19-infected patients and 28 uninfected people in less than 15 min. The T values for the IgG, IgM, and antigen were reported to be 200, 200, and 100, respectively. They showed that all the IgG positives were 100% matched, and only 1 out of 2 were false negatives for the IgM test. Thus, they concluded that their immunoassay had a high specificity and sensitivity and was rapid for the detection of SARS-CoV-2. Gillot *et al* ^114^ reported on a study that dealt with the simultaneous detection of IgG antibodies to 5 different antigens (N, S1, S2, RBD, and NTD) of SARS-CoV-2 via a multiplex immunoassay known as the CoViDiag assay. They showed that for all the antibodies, the specificity of the assay was excellent (99.2 – 100%) and that there were no false positives. They collected samples after 14 days of the initial symptoms and obtained a sensitivity of 92% – 100%. However, they found that after 14 days, there were no statistical differences between patients who were hospitalized and those who were not. Thus, they concluded that further investigation was required for the disease severity to be predicted and for the immune response monitoring of infected patients. Chen *et al* ^46^ studied a lateral flow immunoassay based on surface-enhanced Raman scattering (gap-enhanced Raman nanotags for the ultra-sensitive multiplex detection of anti-SARS-CoV-2 IgM and IgG. They obtained LOD values for IgG and IgM of 0.1 and 1 ng/mL, respectively, and when compared to the LFIA strips that were commercially available, they observed a decrease of 100 times. Moreover, their signal obtained an improvement of 30-fold, showing that their proposed method will have enormous potential in in vitro diagnosis. Kashefi-Kheyrabadi *et al* ^115^ studied the multiple detections of SARS-CoV-2 biomarkers, the S and Orfl1ab genes, using an electrochemical biosensor that had a nanoneedle structure. Their proposed biosensor was able to detect the genes with a high sensitivity (3 and 2 copies/μL for the Orf1ab and S genes, respectively), high selectivity, and could simultaneously detect the Orf1ab and S genes with LOD values of 6.7 and 5.0 ag/μL, respectively, within 1 h. Moreover, they showed that their method was able to detect the genes in both synthetic and clinical samples and was in satisfactory agreement with qRT-PCR results. Masterson *et al* ^116^ reported on a nanoplasmonic label-free biosensor for the simultaneous detection of 10 different SARS-CoV-2 biomarkers, such as the N, E, and RdRp genes, the spike protein subunits, and human antibodies (IgG and IgM) in a single run. In their work, they reported that each biomarker had an LOD lower than 89 aM with excellent specificity. Their proposed method provided a sensitivity (true positive rate) of 100% (80/80), a specificity (true negative rate) of >96% (77/80), a positive predictive value of 98% at 5% prevalence, and a negative predictive value of 100% at 5% prevalence. Their test showed the potential to quantify both IgM and IgG antibodies directly from the plasma of SARS-CoV-2 positive patients without requiring sample preparation and from as little as 10 μL of plasma in 1 h or less. However, they were unable to detect the viral RNA and spike protein in infected patients. Despite this, they concluded that their detection method was sensitive and specific, and by quantifying the SARS-CoV-2 gene sequences and spike protein subunits at an early stage, their approach was able to be more effective in identifying active infection. Song *et al* ^117^ reported on a label-free biosensor based on the upconversion of luminescence nanoprobe/MXene for the detection of both the ORF1ab and N gene of SARS-CoV-2. By measuring the up-conversion fluorescence-enhanced intensity, they were able to achieve a sensitive and rapid (5 min) detection of the genes in one single assay, with LOD values of 914 and 15 pM for the N and ORF1ab genes, respectively. Moreover, their approach allowed for multisite cross-validation; thus, they were able to analyze two gene fragments of the SARS-CoV-2 Omicron variant, which improved the efficiency of detection. Lastly, they confirmed that their results were in strong agreement with the PCR results. Jiang *et al* ^118^ did a study on electrochemical biochips that were based on protein and aptamers for the detection of the SARS-CoV-2 NP and S1, and IgG antibody. Their biosensing chip was shown to have competitive LOD values in the range of fg/mL to ng/mL, a rapid turnaround time (10 min), and good selectivity and specificity toward the S and N proteins. Furthermore, they showed that the aptasensor showed the best sensitivity (1 fg/mL), and their approach showed an accuracy of 100% for discriminating between negative and positive samples.

## Clinical Translation Challenges and Commercialization of SARS-CoV-2 Biosensors

Despite the remarkable progress achieved in the development of SARS-CoV-2 biosensors, the successful translation of these technologies from laboratory prototypes to clinically deployable diagnostic platforms remains a significant challenge. While many biosensors demonstrate excellent analytical performance under controlled laboratory conditions, their performance often differs when applied to complex clinical specimens such as saliva, serum, plasma, or nasopharyngeal swabs. These biological matrices contain proteins, enzymes, salts, and other endogenous compounds that can interfere with biorecognition events or signal generation, potentially reducing sensor sensitivity, selectivity, and overall diagnostic accuracy.

Sample preparation also remains a critical barrier to widespread implementation. Although numerous biosensing platforms have been designed for rapid and point-of-care testing, many still require preprocessing steps such as filtration, dilution, nucleic acid extraction, or viral lysis before analysis. These additional procedures increase assay complexity, extend turnaround times, and limit the practicality of biosensors in resource-constrained settings where minimal user intervention is desirable. Future biosensing strategies should therefore prioritize simplified workflows and direct analysis of clinical specimens without extensive sample manipulation.

Another important consideration is reproducibility. Variations in nanomaterial synthesis, electrode fabrication, surface functionalization, and bioreceptor immobilization can result in significant batch-to-batch differences in sensor performance. Such variability poses challenges for large-scale manufacturing and can hinder the reliability required for routine clinical use. Consequently, the development of standardized fabrication protocols and rigorous quality-control procedures is essential to ensure consistent analytical performance across production batches.

From a commercialization perspective, industrial scalability and standardization remain key requirements. Many reported biosensors rely on sophisticated nanomaterials or multi-step assembly procedures that may be difficult or costly to reproduce on a commercial scale. Furthermore, differences in testing protocols, sample cohorts, and performance metrics across studies complicate direct comparisons between platforms. Establishing standardized validation frameworks, including multicentre clinical evaluations and benchmarking against established diagnostic methods such as RT-PCR and ELISA, will be crucial for assessing clinical utility and facilitating regulatory approval.

Looking forward, successful commercialization of SARS-CoV-2 biosensors will depend not only on achieving exceptional analytical sensitivity but also on demonstrating robustness, reproducibility, affordability, ease of use, and compatibility with existing healthcare infrastructures. Addressing these translational challenges will accelerate the adoption of biosensor technologies and strengthen their role in future infectious disease surveillance, outbreak preparedness, and point-of-care diagnostics.

## Conclusions, Challenges, and Future Perspectives

The outbreak of COVID-19 has had a huge impact on the lives and economic development of people. Due to the continuous emergence of the disease, the number of infected individuals keeps increasing, and the transmission process continues to mutate.^119,120^ This makes it difficult to control and prevent the disease. The year 2021 brought with it high expectations regarding the success of the vaccination campaigns. These efforts were expected to help prevent the spread of the virus. Despite the various steps that were implemented to prevent the spread of the virus, new cases and deaths continued to occur. This called for stricter measures to be taken to minimize the impact of the disease. It is therefore important that the laboratories that are handling this disease are equipped with the necessary equipment and resources to quickly identify and test the virus. This will allow them to prevent the spread of the disease and control its effects. Due to the complexity of the situation, the healthcare system is faced with the challenge of reorganization. Aside from coordinating the various aspects of the pandemic, it must also continuously track other diseases that require follow-up treatment. According to reports, the pandemic has caused various disruptions and postponements in the healthcare system. It is also important that the decisions made regarding the prevention of COVID-19 are taken on a case-by-case basis. This ensures that the benefits of the COVID-19 precautions are more than outweighed by the risks of getting sick. To date, there has been a reduction in the severity of the disease, and the mutation of the virus has led to the possibility that it could coexist with humans in the future.

The comparative analysis of the diagnostic platforms summarized in Tables 1–4 highlights a clear evolution from centralized molecular assays toward ultrasensitive biosensing strategies capable of addressing persistent SARS-CoV-2 viral reservoirs. Table 5 Conventional RT-qPCR and related molecular techniques remain the clinical gold standard due to their excellent sensitivity, specificity, and analytical robustness across multiple viral gene targets. As shown in Table 1, detection limits down to ∼1 PFU/mL^,^ or ∼20 copies/reaction, are routinely achieved, validating their reliability for acute infection diagnosis. However, their reliance on nucleic acid extraction, complex instrumentation, and centralized laboratory infrastructure limits their suitability for real-time, decentralized, and longitudinal monitoring applications. High-throughput sequencing platforms further extend diagnostic scope to genomic surveillance, yet their long turnaround times and bioinformatics requirements restrict their use to specialized settings. Next-generation sequencing platforms further extend diagnostic capability by enabling large-scale screening and genomic surveillance. While these approaches offer unparalleled throughput and variant resolution, their multi-step workflows, long analysis times, and dependence on specialized bioinformatics restrict their use to centralized settings, rendering them impractical for real-time assessment of persistent viral reservoirs. Isothermal amplification and CRISPR-based assays partially address these limitations by reducing assay time and instrumentation complexity. RT-LAMP and CRISPR-Cas systems enable rapid (<45 min) and often visual detection, supporting decentralized testing. Nonetheless, as summarized in Table 1, their sensitivity remains strongly dependent on pre-amplification efficiency and viral load, with reduced performance at high C_t_ values (>30), precisely where persistent or residual viral reservoirs are expected.

**Table 5:** Multiplex Detection of SARS-CoV-2 Biomarkers^a^

Technique	Biomarkers	Detection limit (ng/mL)	Linear range (ng/mL)	Ref.
**Multiplex assay**	N protein	0.5 × 10^–3^	0 – 100	^111^
	S protein	1 × 10^–3^		
**Multiplex assay**	IgG	5	0 – 250	^112^
	IgM	10		
**Multiplex assay**	IgG	1	0 – 1000	^46^
	IgM	0.1		
**Electrochemical biosensor**	ORF1ab	6.7 × 10^–10^	1 × 10^–16^ – 1 × 10^–11^	^115^
	S protein	5 × 10^–10^		
**Nanoplasmonic biosensors**	N, E, RdRp gene, S1 subunit, and IgM and IgG	<1.75 × 10^–9^	0.015 – 9 500	^116^
**Luminesce biosensor**	ORF1ab	3 × 10^–3^	-	^117^
	N protein	0.18		
**Electrochemical** immunosensor/aptasensor	N protein		1 × 10^–6^ – 1	^118^
	S protein	1 × 10^–6^	0.1 × 10^–6^ – 0.1	
	IgG		1 × 10^–6^ – 1	

a N protein = Nucleocapsid protein, S protein = Spike protein, IgG = Immunoglobulin G, IgM = Immunoglobulin M, Open Reading Frame = ORF, RdRp = RNA-dependent RNA polymerase.

Immunoassay-based techniques, including ELISA and lateral flow immunoassays (Table 2), offer important advantages in terms of cost, simplicity, and scalability, particularly for population-level screening and serological assessment. Nonetheless, their sensitivity is often insufficient for early or low-level antigen detection, and performance is strongly influenced by viral load and sampling time, resulting in reduced reliability for monitoring persistent or residual infection. While signal-enhanced formats such as SERS-based ELISA and LFIA partially address these limitations, they still require additional instrumentation and remain constrained by antibody size, orientation, and surface accessibility.

In contrast, electrochemical biosensors and ECL-based immunosensors (Tables 3 and 4) represent a decisive shift toward amplification-free, rapid, and ultrasensitive detection. These platforms routinely achieve fg/mL to pg/mL detection limits with short assay times and minimal sample preparation, making them particularly attractive for probing low-abundance viral antigens or nucleic acids in complex biological matrices. The integration of camelid nanobodies further enhances sensitivity and specificity by enabling higher surface packing densities, improved epitope accessibility, and reduced steric hindrance, which are critical attributes for detecting cryptic viral signatures associated with persistent infection. However, the move from conventional antibodies to nanobodies has yet to make a big impact due to certain challenges that need to be addressed, which ultimately limit their use. Likewise, there is not enough research done on the use of nanobodies as well as biosensing platforms for multiplexed detection of SARS-CoV-2.

Similarly, despite their promise, as stated above, biosensor technologies still face challenges related to large-scale manufacturability, long-term stability, and clinical standardization. Moving forward, no single diagnostic modality is sufficient to address all stages of SARS-CoV-2 infection. Instead, hybrid diagnostic strategies that combine the regulatory robustness of molecular assays with the speed and sensitivity of electrochemical and ECL nanobody-based biosensors are likely to define the next generation of diagnostics. Such convergence will be essential for advancing SARS-CoV-2 detection beyond acute infection toward comprehensive monitoring of persistent viral reservoirs and their long-term clinical implications.

Furthermore, PVR of SARS-CoV-2 has emerged as a critical challenge for long-term disease management, particularly in the context of post-acute sequelae of COVID-19 (PASC or “long COVID”). The existence of these reservoirs in tissues such as the lungs, gut, and immune cells suggests that viral proteins can persist for months after the acute infection. This persistence may contribute to chronic inflammation, immune dysregulation, and prolonged symptoms in affected patients. Consequently, the development of diagnostic tools and biosensors capable of detecting latent or persistent viral reservoirs is essential. Such approaches would enable early intervention, monitor therapeutic efficacy, and potentially guide personalized antiviral treatment strategies to mitigate long-term complications associated with PVR. The integration of advanced biosensing platforms, including nanobody- and electrochemiluminescence-based immunosensors, into clinical workflows may therefore represent a crucial step forward in addressing the challenges posed by PVRs.

Finally, personalized medicine is the future of medicine, as it can help prevent and treat diseases such as COVID-19 and other conditions that claim the lives of millions of people each year. Using electrochemical biosensors, it is possible to create new and personalized approaches to improve the diagnosis and treatment of these conditions.
